# Dividing the Archaeal Way: The Ancient Cdv Cell-Division Machinery

**DOI:** 10.3389/fmicb.2018.00174

**Published:** 2018-03-02

**Authors:** Yaron Caspi, Cees Dekker

**Affiliations:** Department of Bionanoscience, Kavli Institute of Nanoscience, Delft University of Technology, Delft, Netherlands

**Keywords:** the Cdv system, Crenarchaeota, archaeal division, the ESCRT system, membrane remodeling

## Abstract

Cell division in most prokaryotes is mediated by the well-studied fts genes, with FtsZ as the principal player. In many archaeal species, however, division is orchestrated differently. The Crenarchaeota phylum of archaea features the action of the three proteins, CdvABC. This Cdv system is a unique and less-well-studied division mechanism that merits closer inspection. *In vivo*, the three Cdv proteins form a composite band that contracts concomitantly with the septum formation. Of the three Cdv proteins, CdvA is the first to be recruited to the division site, while CdvB and CdvC are thought to participate in the active part of the Cdv division machinery. Interestingly, CdvB shares homology with a family of proteins from the eukaryotic ESCRT-III complex, and CdvC is a homolog of the eukaryotic Vps4 complex. These two eukaryotic complexes are key factors in the endosomal sorting complex required for transport (ESCRT) pathway, which is responsible for various budding processes in eukaryotic cells and which participates in the final stages of division in Metazoa. There, ESCRT-III forms a contractile machinery that actively cuts the membrane, whereas Vps4, which is an ATPase, is necessary for the turnover of the ESCRT membrane-abscission polymers. In contrast to CdvB and CdvC, CdvA is unique to the archaeal Crenarchaeota and Thaumarchaeota phyla. The Crenarchaeota division mechanism has often been suggested to represent a simplified version of the ESCRT division machinery thus providing a model system to study the evolution and mechanism of cell division in higher organisms. However, there are still many open questions regarding this parallelism and the division mechanism of Crenarchaeota. Here, we review the existing data on the role of the Cdv proteins in the division process of Crenarchaeota as well as concisely review the ESCRT system in eukaryotes. We survey the similarities and differences between the division and abscission mechanisms in the two cases. We suggest that the Cdv system functions differently in archaea than ESCRT does in eukaryotes, and that, unlike the eukaryotic case, the Cdv system's main function may be related to surplus membrane invagination and cell-wall synthesis.

## Introduction

**Figure 1:**
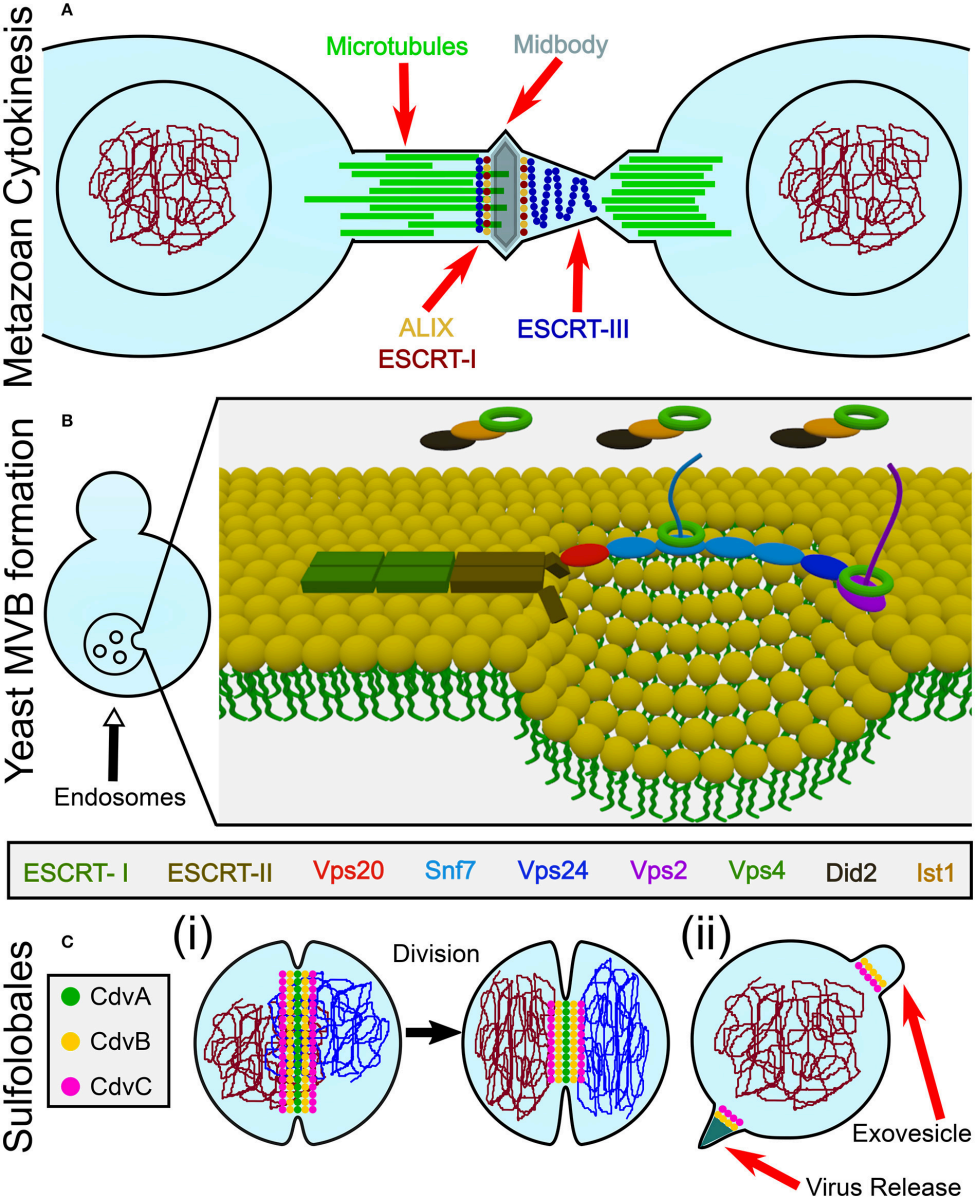
Overview of the biological functions of ESCRT and CdvB proteins. **(A)** In animal cells, ESCRT proteins participate in the last stage of cytokinesis (abscission). **(B)** In Fungi and Animalia, ESCRT proteins are responsible for cargo sorting into the endosomes and for the biogenesis of multivesicular bodies (MVB). For participation of ESCRT proteins in other biological pathways, see the main text. **(C)** In Crenarchaeota, the Cdv system participates in (i) cell division, as well as in (ii) viral release and exovesicle secretion.

Cell division (cytokinesis) is an essential process that, in most biological model systems, is mediated by a proteinaceous cytosolic machinery that binds the plasma membrane. Cytokinesis can be separated into four distinct conceptual stages: (i) localization of the early division components to the division site; (ii) recruitment of later components by the early ones; (iii) application of an ingression force on the membrane, leading to constriction; and (iv) final membrane abscission leading to the daughter cells separation. For example, in a large span of bacterial species, selection of the division site is regulated by the Min protein system and the nucleoid occlusion mechanism that together localize the eukaryote tubulin homolog FtsZ to the cell center (Rowlett and Margolin, 2015). Subsequently, FtsZ recruits late division proteins. The source of ingression force has so far remained unclear but likely includes active cell wall synthesis (while it may also be aided by direct force application by FtsZ itself) (Haeusser and Margolin, 2016; Du and Lutkenhaus, 2017). As of today, it has also remained unclear what drives the final abscission stage (Söderström et al., 2014). Another example of this four steps gradation is found in Metazoa. Here, localization of the division apparatus is mediated by signals that are emitted from the spindle asters and central zone (Barr and Gruneberg, 2007). Subsequently, RhoA is localized to the division site and activates the actomyosin network by indirectly regulating the myosin-II ATPase activity, while the furrow-ingression force is provided by myosin and active actin polymerization (Cheffings et al., 2016). Interestingly, in most animal cells, the end-result of this process is the formation of a thin intracellular bridge between daughter cells (Nähse et al., 2017). At that point, a “NoCut” checkpoint prevents cytokinesis completion until all chromosomal bridges are cleared (Mierzwa and Gerlich, 2014). Subsequently, the Endosomal Sorting Complex Required for Transport (ESCRT) proteins are recruited to the intracellular bridge and mediate abscission, thus completing the cell separation (see Figure 1A).

From a topological point of view, cytokinesis is equivalent to various other membrane remodeling processes such as exovesicle secretion. In all of these cases, a proteinaceous machinery acts from the inner side of the membrane to induce its abscission (accordingly, these processes are called reverse-topology remodeling). In the last few years it became clear that in eukaryotes, the ESCRT system functions as a generalized machinery that cuts the membrane from within and mediates abscission in reverse topology processes. In that sense, ESCRT applies the opposite abscission strategy compared to protein machineries that remodel the membrane from the outer surface (Faini et al., 2013; Daumke et al., 2014). Thus, HIV-1 virus release and the formation of Endosomal Multivesicular Bodies (MVB) became model pathways to study the ESCRT machinery (see Figure 1B).

Relative to the well-studied bacteria and eukaryotes, the process of cell division in archaea is poorly understood. Yet, it follows the same four conceptual division stages, as archaeal cells face the same topological challenges during cell division. In particular, in some archaeal orders that belong to the TACK (Thaumarchaeota, Aigarchaeota, Crenarchaeota, and Korarchaeota) super-phylum, a group of Cdv (Cell division) proteins participates in cell division and equivalent topology membrane-remodeling processes (see Figure 1C). Importantly, some Cdv proteins are homologous to ESCRT proteins (Obita et al., 2007; Hobel et al., 2008). Unlike in Metazoa, however, the Cdv proteins act from early to late stages of the division process. As of today, the exact way in which the Cdv system achieves its function is not yet deciphered.

Often it is suggested that the Cdv system is an evolutionary precursor of ESCRT. Current views regarding the Cdv system largely rely on our understanding of the homologous ESCRT mechanism in eukaryotes. To test this hypothesis, we set out to both extensively review the Cdv function and to critically assess similarities and differences between the ESCRT and the Cdv systems. We will start with a concise review of the division mechanisms of archaea. Next, we review, side-by-side, the repertoire of ESCRT and Cdv proteins and their mutual interactions. We continue by reviewing the *in vitro* reconstitution of ESCRT and Cdv proteins. Following that, we emphasize the role of ESCRT proteins in eukaryotic cytokinesis and the role of the Cdv proteins in the archaeal one. Subsequently, we discuss the different models that were proposed for the ESCRT functioning and critically compare the match between these models and the current knowledge regarding the Cdv system. Finally, we discuss important open questions, and we point out future directions for the Cdv field. From this discussion, we suggest that the Cdv system functions differently in archaea than the ESCRT one functions in eukaryotes. That is, we hypothesize that the archaeal Cdv system is mainly coupled to cell-wall synthesis, like the FtsZ-based bacterial divisome, and/or is responsible for vesicle trafficking into or away from the division site.

Please note that, due to the homology of Cdv and ESCRT proteins, the first group has sometimes been referred to by the corresponding name of the second (which can be quite confusing). To maintain a clear distinction between the two cases, and to stress relevant differences, we will maintain the unique archaeal Cdv terminology. For convenience of the reader, we provide a glossary of Cdv proteins and their relation to the eukaryotic ESCRT proteins in Table 1.

**Table 1:** Glossary of ESCRT and Cdv proteins.

**Complex**	**Yeast name**	**Human name**	**Crenarchaea homolog**	**Asgard homolog**	
CdvA	–	–	CdvA	–	Membrane recruitment
Alix	Bro1	ALIX	–	–	
ESCRT-I	Vps23	TSG101	–	Lokiarch_16740	Adapter and recruitment
	Vps28	VPS28	–	Lokiarch_10170	
	Vps37	VPS37A,B,C,D	–	–	
	Mvb12	MVB12A,B UBAP1	–	–	
ESCRT-II	Vps22	EAP30	–	Lokiarch_37450	
	Vps25	EAP20	–	Lokiarch_37460	
	Vps36	EAP45	–	–	
ESCRT-III	Did2 (Vps46)	CHMP1A,B	CdvB		Membrane remodeling
	Vps2 (Did4)	CHMP2A,B	CdvB1, CdvB2	Lokiarch_37480	
	Vps24	CHMP3	CdvB3		
	Ist1	IST1	–	–	
	snf7 (Vps32)	CHMP4A,B,C	–		
	Vps60	CHMP5		Lokiarch_16760	
	Vps20	CHMP6			
	Chm7	CHMP7			
Vps4	Vps4	VPS4A,B	CdvC	Lokiarch_37470	Dynamical behavior
	Vta1	LIP5	–	–	

*Synonyms of the ESCRT system proteins names in Yeast, Human, and Archaea are outlined. The two classes of ESCRT-III proteins are denoted in yellow and brown, and the Vps2/24 class related Ist1 protein is denoted in dark yellow*.

## Division mechanisms of archaea

For many years, the mainstream thinking regarding archaea put them in proximity to the bacterial domain. Hence, attempts have been made to identify and characterize homologs of the pivotal bacterial division protein FtsZ in archaeal organisms. In the early days, two main archaeal kingdoms, namely the Euryarchaeota and the Crenarchaeota were identified (Woese et al., 1990). Indeed, an archaeal homolog of FtsZ, which in some cases also localized to the division site was identified in several Euryarchaeota species (Baumann and Jackson, 1996; Margolin et al., 1996; Wang and Lutkenhaus, 1996; Nogales et al., 1998; Nagahisa et al., 2000; Poplawski et al., 2000). Subsequent studies showed that FtsZ homologs are found across all classes of the Euryarchaeota kingdom (Makarova et al., 2010). Thus, it was suggested that Euryarchaeota divide via an FtsZ-based mechanism that is similar to the bacterial one.

**Figure 2:**
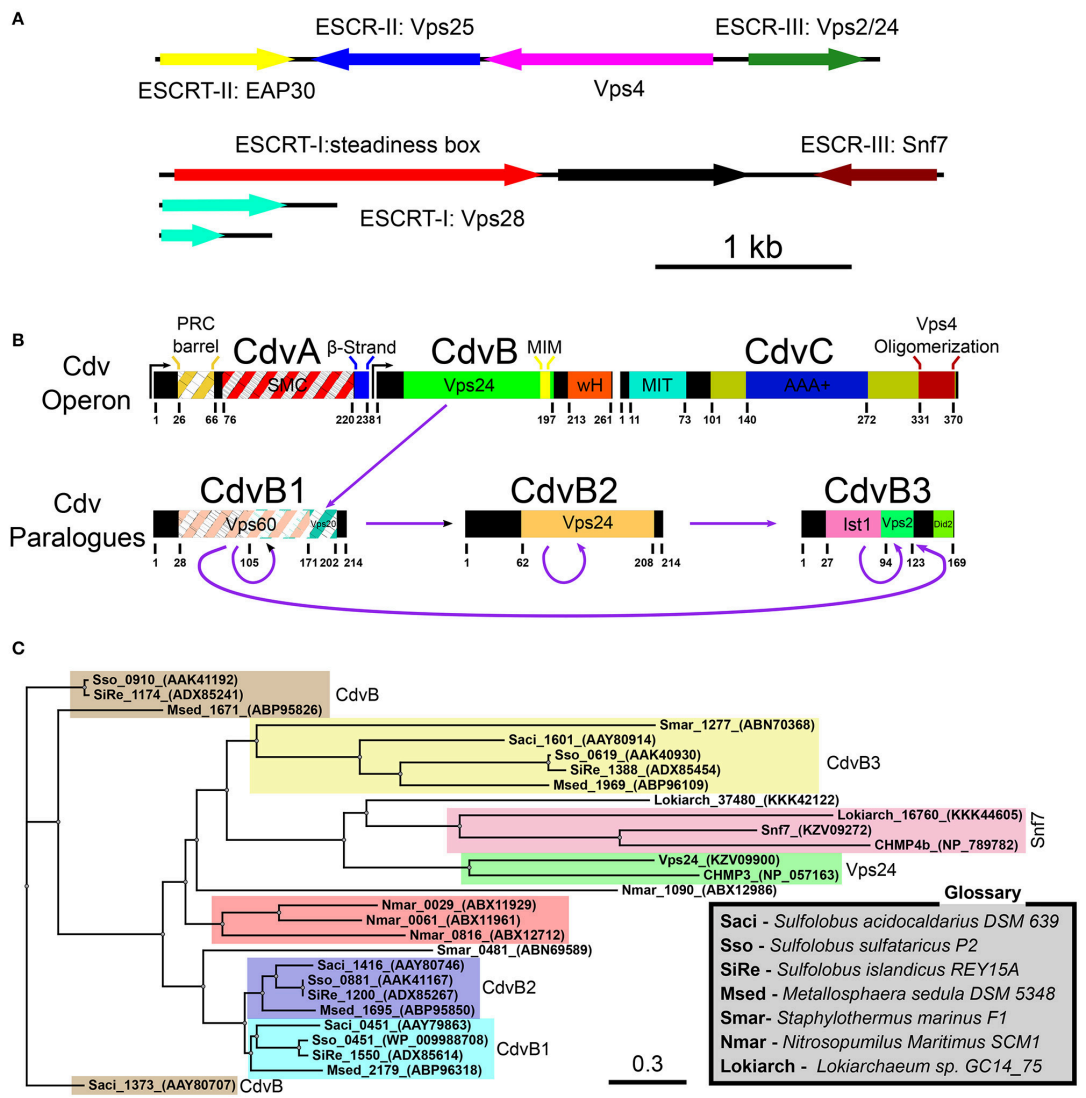
Cdv proteins of archaea. **(A)** Chromosomal distribution of Lokiarchaeota sp. GC14_75 genes that share common features with the ESCRT pathway. ESCRT homologoues (including ESCRT-I and ESCRT-II) are in color. Black, genes that are unrelated to the ESCRT pathway. Based on Spang et al. (2015); Zaremba-Niedzwiedzka et al. (2017). **(B)** Genome organization, domain analysis and interaction network of *S. acidocaldarius* Cdv proteins. The highest homologies of each Cdv protein to the *S. cerevisiae* ESCRT-III proteins are also indicated. Striped boxes indicate homology below statistical significance. Purple arrows, interaction network of *S. acidocaldarius* CdvB proteins. **(C)** Phylogenetic tree of Cdv proteins. Groups of CdvB paralogs are emphasized in colors. Representative members of the two *S. cerevisiae* ESCRT-III protein families as well as the Lokiarchea Cdv proteins are also included. Scale bar, nucleotide substitutions per site.

The current view, however, places the eukaryotes branching point inside the archaea domain so that the TACK super-phylum shares the same phylogenetic ancestor with eukaryotes (Williams et al., 2013). In fact, recently, a deep-sequencing metagenomic analysis inferred the existence of a new archaeal super-phylum that is related to the TACK super-phylum and was named Asgard. It was suggested that Asgard are the archaeal organisms that are closest to eukaryotes (Zaremba-Niedzwiedzka et al., 2017). Interestingly, Asgard cells possess protein signatures that once were thought to be unique to eukaryotes. In particular, they have orthologs for (de)ubiquitination proteins as well as orthologs for proteins that belong to the ESCRT pathway (see Figure 2A and Tables S1, S2 of the Supporting Information; Spang et al., 2015). Since the ESCRT proteins participate in eukaryotes cell division, it can be assumed that their homologs in the Asgard super-phylum are also responsible for cell division. Note, however, that, as of today, none of the Asgard organisms has been cultivated or even isolated, and knowledge regarding their cell biology has been limited to sequencing studies. Thus, no direct evidence connects Asgard ESCRT homologs to cell division.

In contrast, Crenarchaeota organisms, from the TACK super-phylum, especially those from the Sulfolobales order, have become the leading archaeal model organisms. They can be cultivated, and their cell biology is starting to be deciphered (Leigh et al., 2011). While they do not possess homologs of the ftsZ gene, Crenarchaeota organisms do possess homologs to ESCRT proteins—the Cdv proteins. Since ample evidence connects the Cdv proteins to all classical ESCRT pathways, including cytokinesis, the rest of this review will concentrate on Crenarchaeota organisms (and Thaumarchaeota organisms that also possess Cdv paralogs). In particular, references to Cdv proteins will, unless stated otherwise, be to *Sulfolobus acidocaldarius*. For completeness, we mention that homologs of CdvB and CdvC are also found in the Euryarchaeota kingdom (Makarova et al., 2010). However, nothing is known about their cellular function, or whether they participate in cell division. For examples of homology between the *S. acidocaldarius* CdvB and CdvC proteins and their Euryarchaeota counterparts, see Table S3 of the Supporting Information. The fact that CdvB/C appear only sparsely in this kingdom, on top of the fact that Euryarchaea possess functional ftsZ genes, suggests that Euryarchaea Cdv proteins do not play a dominant role in Euryarchaeota cytokinesis.

Interestingly, the Crenarchaeota are further classified into three orders: Thermoproteales, Desulfurococcales, and Sulfolobales. While genes encoding Cdv homologous were identified in the latter two orders, no such genes were identified in the Thermoproteales (Makarova et al., 2010). Instead, it was suggested that Thermoproteales make use of a division system that is based on crenactin, a close homolog of the eukaryotic F-actin (Izorè et al., 2016). However, an identification of the division mechanism of Thermoproteales is still missing, and some experimental evidence suggests that the actin-based division picture is too simplified. For example, in the Thermoproteale *Pyrobaculum calidifontis*, the crenactin is distributed in an extended helical structure that does not localize to the division site (Ettema et al., 2011). By contrast, arcadin 2, which depolymerizes crenactin polymers (Izorè et al., 2016), is localized between segregated chromosomes. This may suggest that, in Thermoproteales, division occurs by destabilization of the cell cortex at the division site while maintaining its rigidity at the poles. In this scenario, the concurrent increase of the rigidity of the cell cortex far away from the division site due to crenactin polymerization, together with destabilization of the crenactin network at the division site, results in a global deformation of cell shape due to energy minimization. In fact, such a mechanism was demonstrated theoretically for lipid-vesicle deformation (Božič et al., 2014) and was suggested as an auxiliary mechanism supporting division in eukaryotes (Wang, 2005). Interestingly, in the Thermoproteale *Pyrobaculum aerophilum* no constriction of the plasma membrane was observed during division (Lundgren et al., 2008), and in *Thermoproteus tenax* a constriction-independent “snapping” mechanism was suggested (Horn et al., 1999). These data suggest that in Thermoproteales division may occur independently of septa formation.

## Overview of the ESCRT and Cdv proteins

To facilitate the discussion about the Cdv system, we will next review the repertoire of Cdv proteins in relation to their ESCRT counterparts. We particularly emphasize here their shared and different structural biology properties.

### The ESCRT pathway

The eukaryotic ESCRT system is composed of five complexes ESCRT-0, -I, -II, -III and VPS4, as well as several associated proteins (see Table 1). It is highly conserved in opisthokont cells (animals and fungi; Field and Dacks, 2009). However, in many non-Metazoa eukaryotes, ESCRT-0 is absent, and while not all components of ESCRT-I were identified, upstream ESCRT elements widely exist (Leung et al., 2008). The abundant existence of upstream ESCRT components in the entire eukaryotic kingdom suggests that the last common universal eukaryotic ancestor already possessed a developed ESCRT machinery. For a recent extensive review about the ESCRT system (see Schöneberg et al., 2017).

Initially identified in the context of MVB formation (Coonrod and Stevens, 2010), the number of biological functions that are assigned to the ESCRT system has increased considerably over the years (see Hurley, 2015; Campsteijn et al., 2016 and references therein). As of today, it includes MVB formation, exovesicles secretion, retrovirus release, cytokinesis, neuronal pruning, plasma membrane healing, nuclear envelope sealing and removal of malfunctioning nuclear pore complexes. In the classical MVB pathway, ESCRT-0 first binds ubiquitinated endosomal membrane proteins that are designated for transport to the lysozyme. Next, ESCRT-0 recruits the ESCRT-I complex, which then recruits the ESCRT-II complex, a complex whose structure best fits a membrane with one concave and one convex curvatures (such as the one that is formed in bud necks; Im et al., 2009; Boura et al., 2012). It was suggested that the ESCRT-II shape is particularly important for the stabilization of narrow membrane necks. Subsequently, ESCRT-II recruits the ESRTC-III complex, which is believed to be the main player in membrane deformation and induces membrane fission through the formation of higher-order polymeric structures. Finally, ESCRT-III recruits the de-ubiquitinated proteins AMSH and Doa4 as well as the Vps4 complex. Vps4, an ATPase and the sole energy-coupled enzyme in the ESCRT pathway, finally disassembles the ESCRT-III proteins from the complex, thus ensuring its turnover. In some cases, ESCRT-III is directly recruited to the membrane by another adapter protein, ALIX.

In yeast, the main component of the ESCRT pathway, the ESCRT-III complex, is composed of 8 protein [6 Vacuolar protein sorting (Vps) proteins, Chm7, and Ist1]. In humans, there are corresponding 12 proteins [11 Charged MVB Proteins (CHMP) and IST1]. Although there are 12 CHMP proteins in humans, several are paralogs (e.g., CHMP4A/B/C), and hence, the human ESCRT-III proteins can also be grouped into eight protein families, parallel to those in yeast. The eight protein families can be further classified into two main classes that share very low sequence homology, Vps2/Vps24/Did2/Ist1 (CHMP2/CHMP3/CHMP1 and IST1), and Snf7/Vps20/Vps60/Chm7 (CHMP4/CHMP6/CHMP5/CHMP7).

### The Cdv system

In Sulfolobales, the Cdv proteins are composed of two groups. The first group includes the cdvA, cdvB, and cdvC genes that are organized in one chromosomal locus. The second group contains three cdvB paralogs, namely, cdvB1, cdvB2, and cdvB3, that are spread at different locations along the chromosome (see Figure 2B). These four cdvB genes are homologs of the eukaryotic Vps2/24/Did2 ESCRT-III class. In addition, the cdvC gene is a homolog of vps4. However, no homologs of the Snf7/Vps20/60 class were identified in Sulfolobales. In addition, cdvA is unique to Sulfolobales, and is not found elsewhere except in Thaumarchaeota and Desulfurococcales. It was suggested that the main function of CdvA is to recruit CdvB to the membrane (Samson et al., 2011). In particular, while the presence of CdvC and CdvB homologs is the definite signature for the existence of a Cdv-like system, CdvA is not generally found in Asgard phylum. Assuming that the main function of CdvA is to recruit CdvB to the membrane, this is understandable, since Asgard organisms possess ESCRT-I/II homologs that can substitute for CdvA. Experimental evidence connects the Cdv proteins to three cellular functions: Exovesicle secretion, viral release, and cell division. In this review, we will mainly concentrate on cell division. For a discussion of the putative role of the Cdv system in exovesicle and viruses release (similar to the ESCRT system), we refer the reader to the Supporting Information. For a detailed recap of the experimental evidence that connects the Cdv system to its different functions in several Crenarchaeota and Asgard organisms, we refer to Tables S1, S2 of the Supporting Information.

Note that the quartet organization of CdvB paralogs in Sulfolobales with four distinct families (CdvB, CdvB1, CdvB2, and CdvB3) is not a universal feature in the TACK super-phylum or even in Crenarchaeota. For example, most of the Desulfurococcales organisms possess only three CdvB paralogs and some organisms possess only two CdvB paralogs (one at the main Cdv locus and the second one at a different location on the chromosome; Makarova et al., 2010). In addition, none of the Desulfurococcales cdvB paralogs belong to the same family as the Sulfolobales cdvB gene. Similarly, the Thermoproteale *Nitrosopumilus Maritimus* possesses four CdvB paralogs, but three out of them form a unique separate family that is not directly related to either the CdvB1/2 or the CdvB3 families. Moreover, the main locus cdvB gene is short (like the Sulfolobales CdvB3), but is not closely related to any Sulfolobales cdvB gene (see Table S2 of the Supporting Information, Figure 2C and Makarova et al., 2010). Thus, a wide variety of CdvB paralogs organization exist in the TACK super-phylum, and it is of interest to study how these different organizations relate to the biological functions of the Cdv system.

Importantly, in contrast to Crenarchaeota and Thaumarchaeota, Asgard organisms such as Lokiarchaeota possess two ESCRT-III-like genes. One belongs to the Vps2/24/Did2 class and is a homolog of CdvB, and one is related to the other ESCRT-III class, that of Snf7/Vps20/60. The existence of proteins from the two ESCRT-III classes in Asgard organisms suggests that the core ESCRT machinery evolved before eukaryogenesis, and that it was followed by ESCRT-III gene duplication for specialization and division of labor. It will be of interest to study how do the different organizations of Cdv proteins contribute to the biological functions of the system.

### The ESCRT-III complex

**Figure 3:**
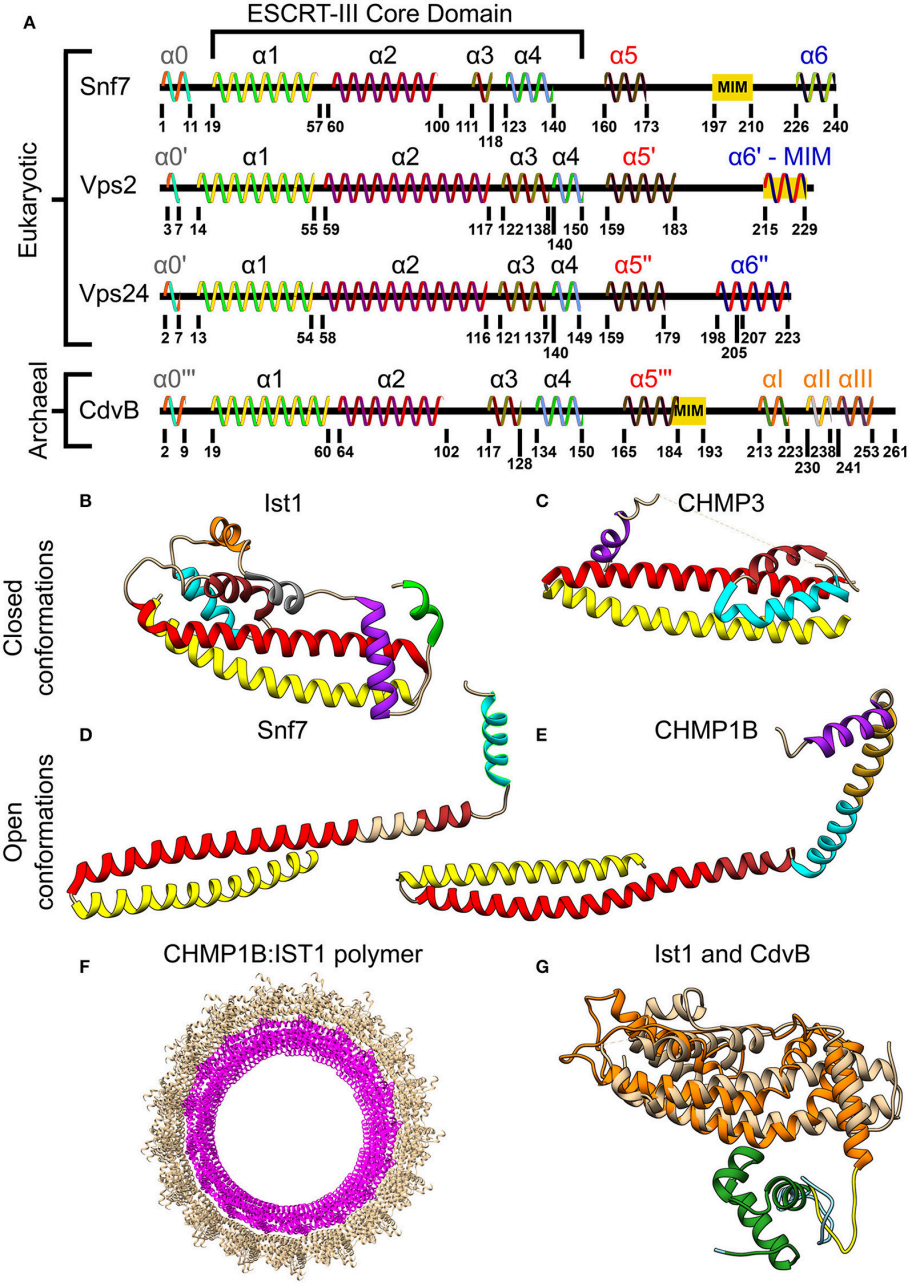
Molecular structure of ESCRT-III and CdvB proteins. **(A)** Secondary structure of several *S. cerevisiae* ESCRT-III (Tang et al., 2015) and *S. acidocaldarius* CdvB proteins. ESCRT-III proteins—helices that are not part of the ESCRT-III core domain are shown with tags. For CdvB, the wH helices are numbered using Greek letters. Since no high-resolution structure of either Vps2 or CdvB exists, the numbering of the helices in these cases is only putative. **(B,C)** Closed conformation of the ESCRT-III proteins. **(B)** IST1 from Cryo-EM (PDB #3JC1) (McCullough et al., 2015). α1, Yellow; α2, Red; α3, Brown; α4, Cyan; α5, Purple; α6, Green. IST1 non-canonical helices are in orange and gray. **(C)** Crystal structure of CHMP3 (Residues 8–222, PDB # 3FRT) (Bajorek et al., 2009b). **(D,E)** Open conformation of ESCRT-III proteins. **(D)** *S. cerevisiae* Snf7 core domain (PDB #5FD9) (Tang et al., 2015). **(E)** CHMP1B from Cryo-EM (PDB #3JC1) (McCullough et al., 2015). Color code for **(C–E)** same as **(B)**. Note that for CHMP1B the interfaces between α2 to α3 and α4 to α5 are only putative. **(F)** Cryo-EM structure of a reconstituted ESCRT-III positive curvature membrane binding ring (PDB ##3JC1). IST1 molecule in tan and CHMP1B in magenta. **(G)** Alignment of *S. acidocaldarius* CdvB Phyre2 based predicted structure and IST1 (PDB #3FRS, residues 1–189; Bajorek et al., 2009b). IST1 is shown in light tan. CdvB core domain is shown in orange, the wH domain in green and the MIT motif in yellow (the rest of the chain in cyan). RMSD distance between 131 atoms 4.916 Å.

Although the two classes of ESCRT-III proteins (Vps2/Vps24/Did2/Ist1 and Snf7/Vps20/Vps60/Chm7) share very low sequence homology, they do share the same secondary structure that is the definite signature of an ESCRT-III protein (see Figure 3A). At the N-terminus of all ESCRT-III proteins, four α-helices are located. Together these four helices form the ESCRT-III core domain (Tang et al., 2015). Two additional, regulatory, α-helices (α5 and α6) are located at the C-terminus. ESCRT-III proteins also share the same overall tertiary structure. In solution, ESCRT-III subunits are found in a “closed” conformation where α5 and α6 fold over the ESCRT-III core domain and inhibit polymerization (Shim et al., 2007; Bajorek et al., 2009b; Tang et al., 2015). Once the auto-inhibition is released (e.g., after binding the membrane), a conformational change transfers the proteins into their “open” form, which enables them to establish higher-order structures (see Figures 3B–E for examples of ESCRT-III proteins in their open and closed conformations). Interestingly, in all ESCRT-III proteins (except Ist1), α1–α3 are highly basic (e.g., pI = 10.71 for Snf7 residues 1-118), while α4–α5 are highly acidic (pI = 3.52 for Snf7 residues 119-240; Babst et al., 2002; Shim et al., 2007). This separation of charge is responsible for the ability of ESCRT-III proteins to bind acidic lipids by creating a highly basic surface. For example, for CHMP3 a basic patch which is conserved from yeast to human was implicated in the membrane binding (Muzioł et al., 2006).

*In vivo*, only 4 proteins are essential for ESCRT-III function (Vps20/Snf7/Vps24/Vps2). The rest of the ESCRT-III proteins act as helper proteins (Ist1, Vps60, and Did2) or are needed for special functions (e.g., Cmp7, in the clearance of nuclear pores). Vps20/CHMP6 is the initiator of ESCRT-III polymerization and recruits Snf7/CHMP4. Snf7/CHMP4 is the major structural protein in the ESCRT-III complex and occupies at least 50% of the complex content (Teis et al., 2008). Vps2/CHMP2 and Vps24/CHMP3, which are recruited to the ESCRT-III complex by Snf7/CHMP4, cap the polymers and recruit the downstream Vps4 complex (Saksena et al., 2009).

**Figure 4:**
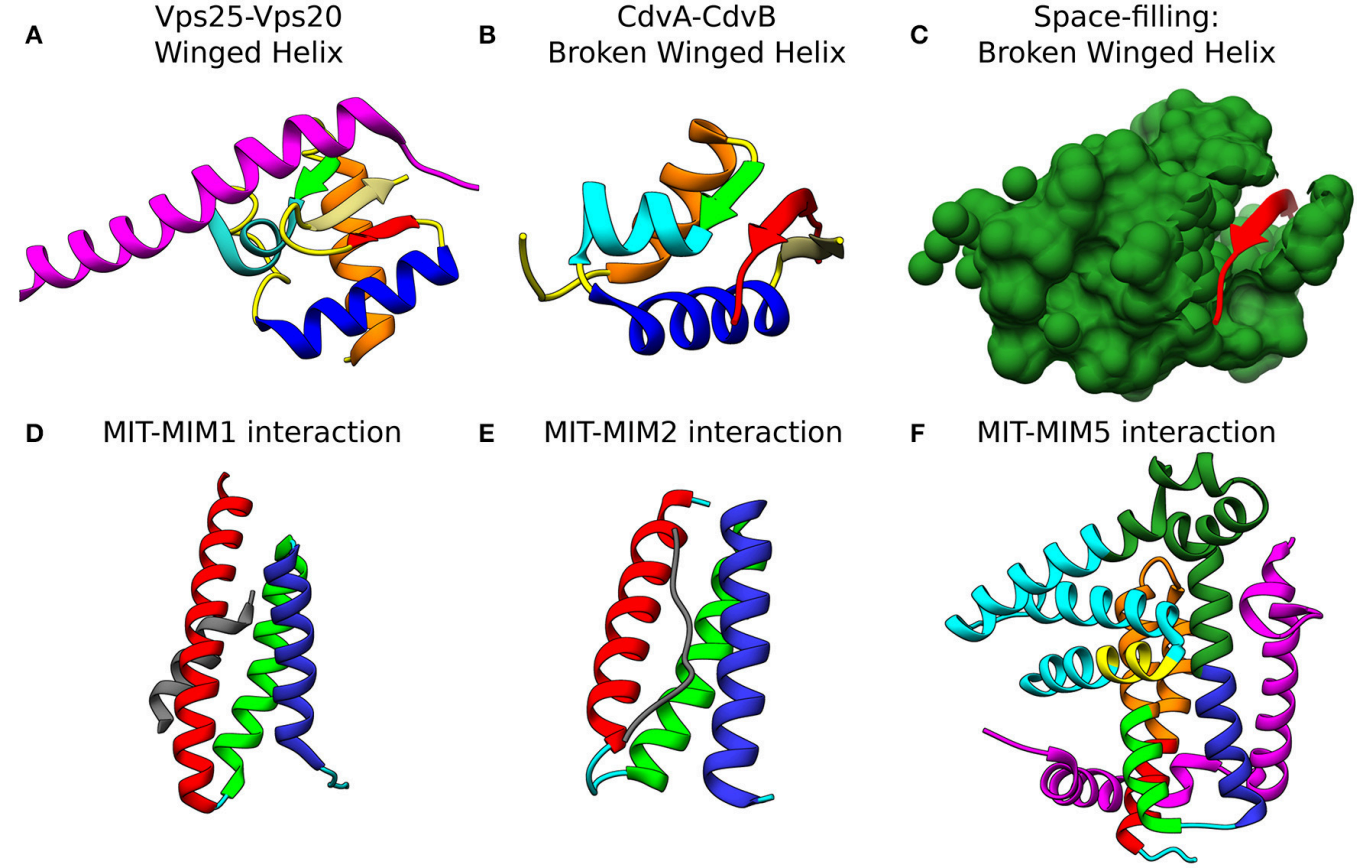
Molecular basis for the interaction of ESCRT-III and CdvB proteins. Top row: wH interactions of ESCRT-III and CdvB proteins with upstream components. **(A)** Crystal structure of the Vps25-Vps20 interaction zone. Vps25 wH domain: α*I*, orange; β1, green; α*II*, cyan; α*III*, Blue; β2, Khaki; β3, Red; unstructured regions, yellow. Vps20 interaction peptide in purple (PDB #3HTU) (Im et al., 2009). **(B)** *S. solfataricus* CdvA-CdvB “broken” wH interaction zone. Color representation—same as in (a) with the appropriate modification of the β-strands numbering (PDB #2XVC) (Samson et al., 2011). E3B peptide of CdvA in red. **(C)** Same as **(B)** with the CdvB wH (light sea green) in space filled representation. Bottom row: ESCRT-III and CdvB interact with downstream components through a MIM-MIT interactions. **(D)** Interaction between the Vps2 MIM1 (gray) and Vps4 MIT domain. (MIT helices; α1, red; α2, green; α3, medium blue; the rest of the chain in cyan). Based on PDB #2V6X (Obita et al., 2007). **(E)** Interaction of *S. acidocaldarius* CdvB MIM2 (gray) with CdvC MIT (PDB #2W2U). Helices colors, same as in **(D)** (Samson et al., 2008). **(F)** Interaction of Vps60 MIM5 (magenta) with Vta1 MIT (PDB #2LUH) (Yang et al., 2012). Helices colors, same as in **(D)**. Homology regions between Vta1 and *S. islandicus* CdvC in orange and green. Homology between Vta1 and *S. islandicus* CdvB1 in yellow. The rest of the Vta1 chain is in cyan.

The recruitment of CHMP6 (Vps20) to the membrane is achieved through its interaction with the winged helix (wH) motif of the ESCRT-II protein EAP20 (Vps25) (see Figure 4A; Im et al., 2009). wH motifs are a subgroup of the helix-turn-helix motifs that are composed of 3 α-helices and 3 β-strands and that, many times, act as transcription factors (Gajiwala and Burley, 2000). For the ESCRT machinery, the interaction between CHMP6 and EAP20 has a *K*_*d*_ of 7 μM. Since ESCRT-II binds the membrane with a nM affinity, this constitutes an efficient pathway to initiate ESCRT-III polymerization.

### The CdvB paralogs

As mentioned above, CdvB and its paralogs belong to the Vps2/24/Did2 protein class (see Figure 2B, and also Figure S1 of the Supporting Information for a full homology repository between the *S. acidocaldarius* CdvB paralogs and *S. cerevisiae* ESCRT-III proteins). Like all other ESCRT-III proteins, they are predicted to share the same overall ESCRT-III secondary structure organization, however, with some differences (Figure 3A). In CdvB, the four ESCRT-III core α-helices are present, as well as the auto-inhibitory helix α5, but CdvB lacks the last terminal helix α6. A tertiary structure prediction program that is based on sequence homology and alignment (Phyre, see Kelley and Sternberg, 2009) predicted a CdvB 3D structure that is highly similar to that of Ist1 in the “closed” state (see Figure 3G). However, the N-terminus of CdvB is less basic than that of ESCRT-III proteins, and the C-terminus is only mildly acidic (residues 1–116 pI 10.23; residues 117–212 pI 5.17). This lower ionic charge of the CdvB N-terminal end is probably responsible for CdvB inability to bind the membrane directly. In particular, the basic patch in CHMP3 that was implicated in the membrane binding has, in fact, an acidic pI in CdvB, rendering it unfitted for membrane interaction (Samson et al., 2011). At the far end of CdvB C-terminal end, a wH domain is located, which was implicated in its interaction with CdvA as is discussed below. This wH domain is slightly more acidic than the rest of the C-terminus part (residues 213–261, pI 4.12).

CdvB1 and CdvB2 form two closely related protein subfamilies. They are shorter than CdvB and lack the wH domain. While the N-terminal half of CdvB1 and CdvB2 has a pI similar to CdvB, the C-terminus part of CdvB1 and CdvB2 is somewhat more acidic (CdvB1 residues 117–214 pI 4.06, CdvB2 residues 117–219 pI 4.3). Interestingly, unlike for CdvB, the peptide of CdvB1 and CdvB2 that is a homolog to the membrane binding patch on CHMP3, is somewhat basic (pI 9.8 compare to pI 11.7 in CHMP3). This might suggest that CdvB1 and CdvB2 can bind membrane directly, though less efficient than CHMP3. However, currently, there is no experimental evidence for such an interaction.

In contrast to Sulfolobales CdvB, the Asgard ESCRT-III homologs proteins do share the fundamental ESCRT-III property of a highly basic N-terminus moiety and a highly acidic C-terminus moiety (Lokiarch_37480: residues 1–120 pI 11.12, residues 121–209 pI 3.23; Lokiarch_16760 residues 1–122 pI 11.06, residues 123–218 pI 3.34). In particular, the Lokiarch_37480 peptide that is homolog to the CHMP3 membrane binding patch is even more basic in Lokiarch_37480 than in CHNP3 (pI 12.2). Hence, it will be interesting to study if Asgard ESCRT-III homologs can bind the membrane directly, and if they do, what is the implication on their function.

A yeast two-hybrid system has predicted an intricate network of interactions between the CdvB paralogs, where CdvB1/2/3 each interact with itself as well as with the other two CdvB paralogs (see Figure 2B) (Samson et al., 2008). In addition, CdvB interacts with CdvB1. It is interesting to note that CdvB3, the shortest CdvB paralog that can probably accommodate only the ESCRT-III core domain (helices α1–α5), shares homology with the ELYC domain of Ist1. The ELYC domain is involved in the interaction between the human IST1 homolog and CHMP1 (Dimaano et al., 2008). Thus, this homologous domain might enable the interaction between CdvB3 and CdvB1/CdvB2. To the best of our knowledge, this is the first time that such a homology is noted.

### CdvA and its interaction with CdvB

As mentioned above, CdvA is unique to the TACK superphylum. It does not share significant homology with any known protein families. The only significant homology that we were able to identify outside the TACK superphylum is to two distant proteins in *Thorarchaeota*, an organism from the Asgard superphylum. CdvA probably has a tripartite structural organization. Secondary structure prediction suggests the existence of a β-strand-rich domain at its N-terminus, which is followed by a long α-helix-rich domain. The latter domain occupies the major part of the protein sequence and is followed by an unstructured region. Several non-significant homologies were previously noted between CdvA and other proteins. For example, the β-strand-rich moiety was suggested to form a PRC barrel domain (Samson et al., 2011) and the α-helix-rich moiety was suggested to belong to the lamins, golgins and cingulin protein of eukaryotes (Lindås et al., 2008). In the NIH database, the α-helix-rich domain is suggested to belong to the Structural-Maintenance of the Chromosome (SMC) fork B family for *S. acidocaldarius* and to the BAR domain family for *Metallosphaera sedula*. Indeed, we have noted some homology between the CdvA α-helices-rich domain and the SMC protein of *B. subtilis*. In addition, we noted some non-significant homology between the *S. acidocaldarius* α-helices-rich domain and one of the helices of ALIX. Overall, this set of non-significant homologies probably only represents the double function of CdvA as a membrane and a DNA binder as discussed below.

At the C-terminus of CdvA another short β-strand is located which was named E3B. The E3B peptide is responsible for the CdvA-CdvB interaction through its binding to the CdvB wH domain with a *K*_*d*_ of 6 μM (similar to the ESCRT-II-Vps20 interaction affinity) (Samson et al., 2011). Interestingly, while Vps20 binds an exterior surface of the ESCRT-II wH domain (see Figure 4A), in the CdvB-CdvC interaction interface, the wH domain is broken, and one of the β-strands is missing. In that case, the broken wH domain is supplemented by the CdvA E3B peptide that together form a full wH domain, albeit with three parallel β-strands (see Figures 4B,C). This forms a unique wH domain interaction that was probably developed in Crenarchaeota. Since CdvA can bind the membrane, as is discussed below, it is customary to model CdvA as the recruiter of CdvB to the membrane. Indeed, from all CdvB paralogs, only CdvB possess a wH domain.

### The Vps4 complex

**Figure 5:**
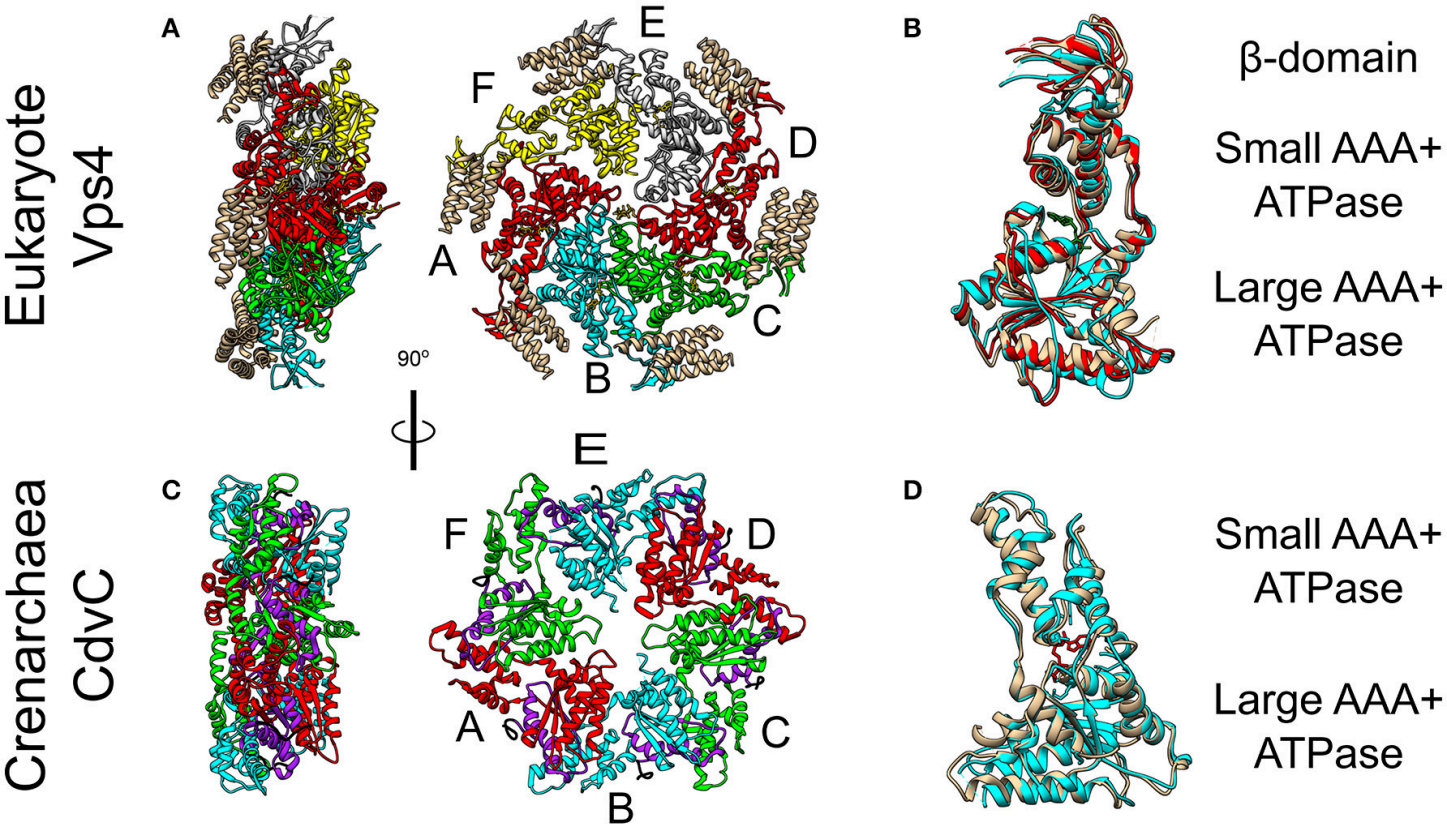
Crystal Structure of the Vps4 and CdvC proteins. **(A)** Crystal structure in side (left) and front (right) views of the yeast Vps4 hexamer together with the VSL domain of Vta1 (tan). Protomers are marked as A–F. Equivalent monomers share color. Gray protomers bind either ADP or ADP+Pi. The yellow protomer is empty (PDB #5UIE) (Monroe et al., 2017). **(B)** Alignment of monomers F (tan), E (red) and C (cyan). F to C protomers alignment—RMSD between 300 atom pairs is 3.262 Å. **(C)** Crystal structure side (left) and front (right) views of an empty *M. sedula* CdvCΔMIT hexamer (PDB #4D80) (Caillat et al., 2015). Identical protomers share the same color. The P-Loops NTPase domains (residues 105–156) are highlighted in purple. The N-terminus of every chain is shown in black. **(D)** Alignment of one protomer from **(C)** in tan with *M. sedula* CdvC bound to ADP (PDB #4D82) in cyan. RMSD between 257 atom pairs is 2.717 Å.

The Vps4 complex couples the recurring action of the ESCRT-III complex to energy expenditure through its ATPase activity. Thus, Vps4 sets the directionality of the membrane remodeling pathway. In yeast, in the ATP-bound form, the Vps4 complex is a hetero-hexamer of Vps4 and the Vps4 cofactor Vta1 (see Figure 5A) (Monroe et al., 2014). In fact, ATP is necessary for the Vps4 oligomerization. Each Vps4 monomer is composed of a Microtubule Interacting and Trafficking (MIT) domain, a large ATPase subunit, a small ATPase subunit, and a β-domain (see Figure 5B). Vta1 binds the β-domain and stimulates Vps4 ATPase activity (Azmi et al., 2008). Multiple experiments have recently shown that although Vps4 appears as a hexamer, it does not form a closed hexameric ring (Monroe et al., 2017; Su et al., 2017; Sun et al., 2017). Instead, it possesses a helical form where ATP hydrolysis and ADP release result in structural modifications of the interfaces between the monomers (see Figure 5A). To pull out and recycle ESCRT-III subunits, Vps4 first binds MIT Interaction Motives (MIMs) of the ESCRT-III subunits. This reveals part of the α5 helix of the ESCRT-III subunit for a high-affinity interaction (*K*_*d*_ ~ 2 μM) with loops in the Vsp4 central pore (Han et al., 2015). In addition, the Vps4-ESCRT-III binding also stimulates the ATPase activity of Vps4. Consequently, the helical form of Vps4 allows it to “walk” along the ESCRT-III polypeptide chain and pull it out of the ESCRT-III complex as a thread through the Vps4 complex central pore (Yang et al., 2015). Interestingly, in addition to the Vps4 monomers, Vta1 also possesses 2 MIT domains. Thus, overall, the Vps4 complex exposes 18 MIT domains, which interact selectively with different ESCRT-III subunits.

### CdvC

CdvC shares both a high sequence (37% identical, 52% positive over 345 amino acids) and high structure (*S. solfataricus* RMSD 1.62 Å over 237 pairs of *C*^α^ atoms) similarities to Vps4 from *S. cerevisiae* (Monroe et al., 2014). However, archaea lack the Vta1 cofactor. In addition, two small structural differences exist between CdvC and the Vps4 protein. First, CdvC lacks the Vps4 major β-domain that connects the two parts of the small AAA+ ATPase subunit, and which is responsible for the binding of Vta1 (see Figures 5B,D and Monroe et al., 2014; Caillat et al., 2015). Instead, for CdvC, the two parts of the small subunit are connected by a well-ordered short loop. Second, in its empty form, the large AAA+ ATPase subunit of CdvC contains only 4 β-strands in comparison to 5 in its eukaryote counterpart. The small fifth β-strand (annotated as β′) that is located at the beginning of the large subunit, next to the MIT domain, is unstructured in the empty state of CdvB. Only in the ADP-bound form of the protein, this moiety becomes structured into a short β′-strand and an α-helix.

Crystal structure studies as well as size exclusion chromatography of the Crenarchaeota *M. sedula* CdvC, showed that it oligomerizes into hexamers, similar to Vps4 (see Figures 5A,C; Monroe et al., 2014). However, unlike Vps4, CdvC can form a hexameric ring even without ATP. In the empty form, the hexameric ring shows a 3-fold symmetry with 3 pairs of identical protomers that together form a planar asymmetric structure (see Figure 5C). When ADP binds CdvC, a 23° rotation between the two ATPase domains is induced. This rotation is a manifestation of the structural flexibility of the protein that is necessary for its proper function. Also in its tertiary structure, like in its secondary structure, there are several differences between CdvC and Vps4. First, ADP bound to CdvC occupies a position that is similar to that of ATPγS when it binds the yeast Vps4. Second, ADP bound to Vps4 penetrates less into the binding pocket than ADP that binds CdvC. In addition, isothermal titration calorimetry showed that CdvC binds with a similar affinity six ATP molecules with *K*_*d*_ = 3.3 μM, while for ADP, five molecules had a similar affinity of 5 μM, and one ADP had a high affinity of 0.4 μM (Caillat et al., 2015). For Vps4 in yeast, however, only a negligible amount of bound ADP was detected in a purified non-hydrolyzable version of the hexamer, so the *K*_*d*_ of ADP is probably much lower than that of ATP (Sun et al., 2017). Moreover, although biochemical assays showed that it hydrolyzes ATP in the hexameric active form (Moriscot et al., 2011; Caillat et al., 2015), in contrast to Vps4, CdvC's ATPase activity is only marginal at 37°C and becomes substantial only at high temperatures [16 $\frac{ATP}{min}$ at 60°C (Caillat et al., 2015), compared to 45 $\frac{ATP}{min}$ for Vps4-Vta1 at 30°C (Azmi et al., 2008)]. These differences may suggest a somehow different ATP cycle for CdvC action than for the yeast Vps4.

### The interaction between ESCRT-III and Vps4

Similar to their MIM-MIT based interactions with Vps4, ESCRT-III proteins utilize their MIM peptide for the interactions with other downstream components such as the de-ubiquitinated protein AMSH (Hurley and Yang, 2008; Shestakova et al., 2010). Different MIM sequences in various ESCRT-III proteins and different MIT domains in various down-stream components permits differential binding and division-of-labor specialization. In general, the MIM peptides are located at the C-terminal domain of the ESCRT-III subunits (see Figure 3A). As of today, at least five types of MIM-MIT interactions were identified (Yang et al., 2012). Two out of these are being utilized for the ESCRT-III interactions with Vps4 (see Figures 4D,E). MIM1 peptide, found in proteins from the Vps2/Did2 class, forms a α-helix and complements the interface between α2 and α3 of the MIT domain. The characteristic feature of MIM1 is the formation of leucine-based hydrophobic interactions and charged amino acids based salt bridges with the MIT domain. In contrast, the MIM2 peptide binds the opposite side of the MIT domain, between α1 and α3, and is found at the C-terminal domain of proteins from the Snf7/Vps20/Vps60 class. A consensus sequence of the MIM2 motif based on archaeal and eukaryotic organisms was suggested to be: ϕ_1_*P*_1_*xϕ*_2_*P*_2_*xxP*_3_ϕ_3_*P*_4_, where *P* is proline, *x* a polar residue and ϕ a hydrophobic residue (Kojima et al., 2016). Thus, the characteristics feature of the MIM2 motif is a high enrichment of proline residues. Exceptionally, Ist1 possesses both MIM1 and MIM2 motifs, showing that proteins from the Vps2/24/Did2 class can also possess an MIT2 motif.

The MIM-MIT interactions exhibit a wide span of binding affinities, from a high binding efficiency (1–2 μM, to tens of μM), to a very low binding efficiency (more than 100 μM) (Obita et al., 2007; Stuchell-Brereton et al., 2007; Kieffer et al., 2008; Bajorek et al., 2009a). In particular, CHMP4, the main constituent of the ESCRT-III complex binds Vps4 with a very low binding affinity, while Vps2, the essential factor for the recruitment of Vps4 to the ESCRT-III structure binds Vps4 with an affinity of about 30 μM. In addition, IST1 binds VPS4 with a very high binding efficiency (about 1 μM), which enables it to bind VPS4 in the cytoplasm and recruit it to the ESCRT-III complex thus assuring the efficient function of the ESCRT pathway (see Figure 1B). In contrast, proteins with lower binding affinities have to be incorporated into the ESCRT-III complex before they can bind Vps4 efficiently. Thus, this broad span of interaction strengths implies functional consequences.

### The interaction between CdvB and CdvC

Similar to the ESCRT-III-Vps4 interaction, also the CdvB-CdvC interaction is mediated through an MIM-MIT binding. Interestingly, although CdvB is a homolog of the Vps2/24/Did2 class, it does not possess an MIM1 motif. Instead, it possesses an MIM2 peptide at the end of the C-terminal region, immediately after the α5 (Figure 4E). CdvB thus exhibits mixed characteristics. On the one hand, it possesses a Vps2/24/Did2 core domain. On the other hand, it possesses an Snf7/Vps20/60-family MIM2 peptide for its interaction with CdvC.

For CdvB, the MIM2 peptide sequence is: $\underline{\underline{\text{RE}}\text{LL}}\text{P}\underline{\underline{\text{E}}\text{L}}\text{P}\underline{\underline{\text{H}}}\text{PP}$ (underbar, hydrophobic; double underbar, acidic or basic). Thus, the main characteristic of the MIM2 motif, namely an enrichment of plorine residues is maintained. Residues 177-261 of CdvC, which contain this motif, bind the CdvC MIT domain with a *K*_*d*_ ≈ 30 μM, thus situating the CdvB-CdvC interaction on the middle range of ESCRT-III-Vps4 binding efficiencies, similar to the Vps2-Vps4 interaction strength (Samson et al., 2008).

By contrast to CdvB, C-terminus peptides from *S. acidocaldarius* CdvB1 and CdvB2 show only a marginal binding affinity to the MIT domain (*K*_*d*_ > 100 μM). This is consistent with the fact that CdvB1 and CdvB2 lack the end part of the proline-rich MIM2 motif ($\underline{\underline{\text{KEK}}\text{F}}\text{P}\underset{˜}{\text{S}}\underline{\text{L}}\text{P}\underline{\text{AA}}\text{G}$ and $\underline{\underline{\text{KEK}}\text{F}}\text{P}\underset{˜}{\text{S}}\underline{\text{L}}\text{P}\underset{˜}{\text{S}}\underline{\text{FA}}$ respectively; wavy underbar - polar). Thus, in *S. acidocaldarius*, CdvB is probably the sole recruiter of CdvC *in vivo*. However, this picture was different in *S. islandicus*, as here a yeast two-hybrid system detected interactions also between CdvB1 or CdvB2 with CdvC (Liu et al., 2017). This difference in the CdvC interaction repertoire probably has a causal effect on the mechanisms of action during division, as is discussed below.

We would like to suggest a structural hypothesis that may explain this different binding profiles of CdvB1-CdvC in these two species. The Vta1 ESCRT-III interacting domain is composed of two tandem MIT domains that are packed one against each other at a 90° organization. Vta1 interacts with Vps60 through a unique MIM5-MIT interaction mode (Yang et al., 2012). In that case, two interfaces are implicated in the interaction, one between helix 1 and helix 3 of the MIT domain and a separate one with helix 1 of Vta1 MIT (see Figure 4F). We noticed some homology between the Vta1 MIT domain and CdvC. However, while for *S. acidocaldarius* the sequence homology is low and the homologous region is continuous and is part of the ATPase domain, for *S. islandicus* the homologous sequence is divided into two regions. One of these regions is part of the CdvC MIT domain. Interestingly, in addition, CdvB1 possess a short α-helix that is also a homolog to the Vta1 MIT domain, and this α-helix is involved in the packing of the two Vta1 MIT domains one against the other. This suggests that for *S. islandicus*, the less-proline-rich MIM2 motif may be supported by an additional interaction between the CdvB1 protein body and the CdvC MIT domain, in the same fashion as in Vta1 MIT-MIT packing. If that is the case, this will constitute an additional class of MIM-MIT interaction. It will be interesting to test this hypothesis experimentally.

## *In vitro* reconstitution of Cdv and ESCRT–III proteins

### Higher-order structures formed by ESCRT-III proteins

The hallmark of ESCRT-III proteins is their ability to form higher-order structures such as domes, filaments or spirals. Although the exact membrane-remodeling mechanism of ESCRT-III is still under intensive debate (Chiaruttini and Roux, 2017; Schöneberg et al., 2017), it is generally believed that the key for the membrane deformation and abscission depends on the formation of these ESCRT-III structures.

**Figure 6:**
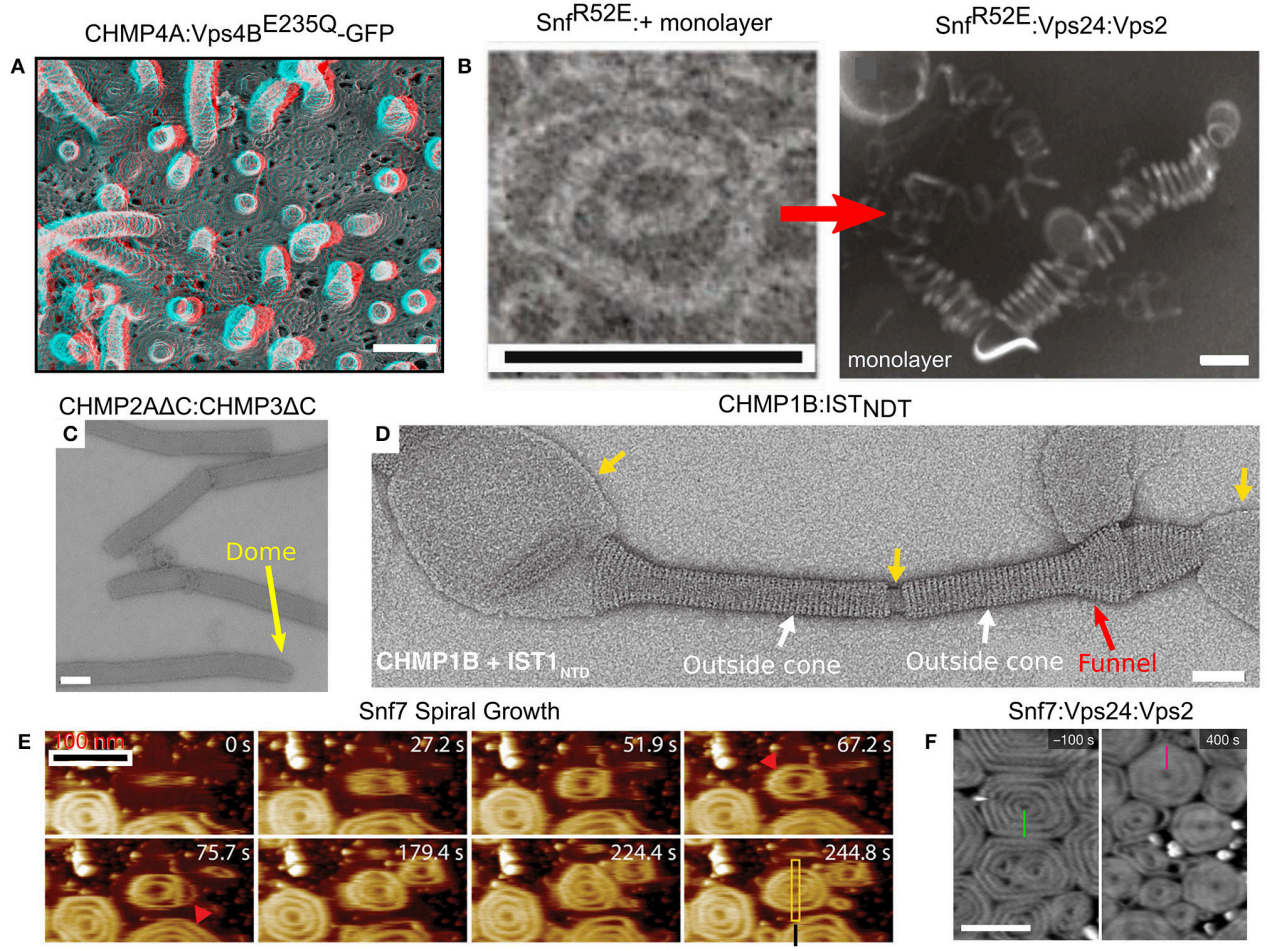
Membrane remodeling by ESCRT-III proteins. **(A)** Protrusions and spirals on the plasma membrane as result of an overexpression of CHMP4A:Vps4B^*E*235*Q*^-GFP in COM-7 cells. Scale bar 100 nm. **(B–F)** *In vitro* reconstituted ESCRT-III polymers. **(B)** Rings and helices: typical 2D Snf^*R*52*E*^ rings and Snf^*R*52*E*^:Vps24:Vps2 3D helices (formed when a lipid monolayer is present). Scales bar 100 nm. **(C)** Tubes of CHMP2AΔC:CHMP3ΔC. Scale bar 100 nm. The dome structure at the tip of the tubes (yellow arrow). **(D)** Cones and funnels: CHMP1B:IST_*NTD*_ coating a lipid vesicle to form an outside sheath. Cf. also Figure 3F. Scale bar 50 nm. **(E)** Growth of Snf7 spirals (red arrowheads) on a supported lipid membrane over time, measured in high-speed liquid AFM. Scale bar 100 nm. **(F)** Pre-formed Snf7 spirals on a supported lipid bilayer (left) are flattened and shrink upon Vps24 and Vps2 addition. Scale bar 200 nm. **(A)** Is reproduced from Hanson et al. (2008). **(B)** is reproduced from Henne et al. (2012). **(C)** Is reproduced from Lata et al. (2008). **(D)** Is reproduced from McCullough et al. (2015). **(E)** Is reproduced from Chiaruttini et al. (2015). **(F)** is reproduced from Mierzwa et al. (2017). All panels are reproduced with permission.

One of the most impressive *in vivo* examples of ESCRT-III polymers appeared when CHMP4A or CHMP4B were over-expressed (Hanson et al., 2008). Under this condition, both proteins formed highly ordered arrays of curved filaments at the plasma membrane. When, in addition, the dominant-negative Vps4B^*E*23*Q*^ was expressed, the CHMP4A filaments created an extensive array of protrusions that bulged out of the plasma membrane (see Figure 6A). The diameter of the protrusions was about 100 nm, and they contained highly dense CHMP4A spiral filaments. Similarly, depletion of VPS4A/B and over-expressing of CHMP2B resulted in the formation of plasma membrane protrusions that could be as long as tens of microns (Bodon et al., 2011). These protrusions were filled with helical CHMP2B filaments throughout their entire length. In fact, ESCRT-III spiral polymers were also observed on the plasma membrane upon Vps4 deletion, without the need for protein over-expression (Cashikar et al., 2014). Thus, members from either of the two ESCRT-III classes assemble on the plasma membrane and can lead to the formation of long protrusions *in cellulo*.

Many ESCRT-III proteins also form complex structures *in vitro*. For example, Snf7 proteins, with a mutation that removed the auto-inhibition, formed flat spirals and ring-like structures when incubated together with a lipid monolayer (see Figure 6B; Henne et al., 2012). When the Snf7 mutant was incubated together with Vps24, Vps2 (molar ratio 2:1:1) and a lipid monolayer, 3D coiled-helical structures were formed (see Figure 6B). The average diameter of these 3D helices was about 85 nm, approximately the size of an endosome-bud neck. Not only did Vps24 and Vps2 determine the geometrical pattern of Snf7 polymers, but they also reshaped preformed flat Snf7 spirals into the 3D helices, suggesting a regulation mechanism for ESCRT-III membrane-remodeling.

By themselves, members of the Vps2/24/Did2 class tend to form long rigid tubes or long linear/branched protofilaments, but not spirals or rings (Bajorek et al., 2009b). For example, Vps24 form a network of branched long (hundreds of nm) helical protofilaments with a typical diameter of 15 nm (Ghazi-Tabatabai et al., 2008), while CHMP2AΔC:CHMP3ΔC, both lacking the auto-inhibition region, formed hundreds of microns long tubes with a width of about 40 nm. In some cases, these tubes ended up with a dome-like structure (see Figure 6C; Lata et al., 2008). Similar structures were also observed for CHMP2A:CHMP3ΔC (Effantin et al., 2013). Yet, under certain conditions, proteins from the Vps2/24/Did2 class can also form coil-like polymers or rings (for example, CHMP2AΔC polymers; Lata et al., 2008; Effantin et al., 2013).

It is interesting to note that the CHMP2AΔC:CHMP3ΔC tubes expose their outer side for interaction with a membrane while Vps4 interacts with the inner surface of the tubes. This arrangement is consistent with the expected topology of the ESCRT-III machinery. By contrast, CHMP1B formed cones and funnels alone or with the N-terminus domain of IST1, but these structures wrapped around the outside surface of vesicles (see Figure 6D). Thus, the CHMP1B structures exhibit an opposite topology to the regular one of the ESCRT machinery (McCullough et al., 2015). High-resolution cryo-EM showed that these CHMP1B:IST1_*NTD*_ tubes consisted of two layers. The inner layer was composed of CHMP1B monomers in the “open” conformation. The outer layer consisted of IST1 in a “closed” conformation (see Figure 3F).

In spite of the fact that various proteins from both ESCRT-III classes assemble into higher order structures, it is believed that Snf7/CHMP4 spirals are the main player in the membrane deformation. By its nature, a spiral does not have a constant curvature. As the spiral ring grows bigger, its curvature grows smaller. Measurements on Snf7 polymers suggested a preferred diameter of about 35 nm (Shen et al., 2014). When Snf7 formed spiral polymers on a supported lipid bilayer, it nucleated from a ring with a typical diameter of about 25 nm (Chiaruttini et al., 2015). As additional turns are added to the spiral, the innermost rings were compressed to about 17 nm and outer rings continued to grow. In addition, the spiral structure evolved into a polygon-like shape, probably to accumulate the stress induced from the non-ideal curvature of the spiral rings (see Figure 6E). These data suggest the Snf7 spirals acts as a tensed spring that deform the membrane by releasing its tension.

Interestingly, when Vps2 or Vps24 were added to Snf7 spirals on a supported lipid bilayer, they inhibited the growth of the Snf7 spirals by bundling with them to form co-filaments with a typical width of 15 nm (Mierzwa et al., 2017). In addition, the Snf7-Vps2-Vps24 polygons compacted to a disc-like structure (see Figure 6F). Addition of Vps4 to the Snf7-Vps24-Vps2 spiral-disks resulted in a massive rearrangement of the network. Pre-existing spirals depolymerized and reduced their size, and new spirals were formed at the expense of the pre-existing ones. Thus, Vps4 confers dynamical-behavior capabilities to the ESCRT-III polymers.

Two of the most impressing examples for the ability of ESCRT-III proteins to remodel membranes *in vitro* occurred when several ESCRT-III proteins were incubated together with lipid vesicles. In the first case, when the four ESCRT-III core proteins were incubated with small unilamellar vesicles, inward-facing buds were produced in a Vps4-independent manner (Saksena et al., 2009). In the second case, incubating the ESCRT-III core proteins with giant unilamellar vesicles resulted in the formation of completely encapsulated vesicles, similar to the MVB, again in a Vps4 independent manner (Wollert et al., 2009). Adding the ESCRT-0/I/II proteins to the giant liposomes *in vitro* assay, enabled Wollert et al. to obtain completely encapsulated vesicles even at a physiologically relevant concentration of the ESCRT-III proteins (Wollert and Hurley, 2010).

### Reconstitution of Cdv proteins into higher-order structures

**Figure 7:**
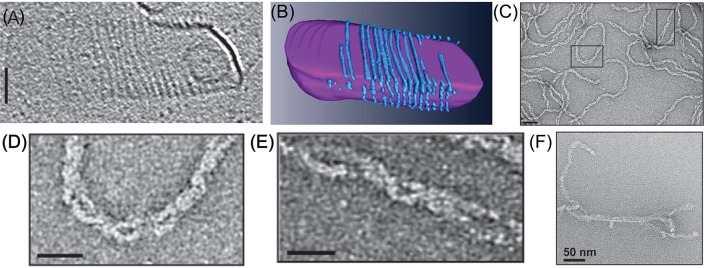
*In vitro* reconstitution of Cdv protein. **(A)** Cryo-EM microscopy of CdvA from *S. acidocaldarius* that polymerize on the other surface of a liposome made from tetraether polar lipid fraction **(E)**. Scale bar - 50 nm. **(B)** 3D reconstruction of the CdvA filaments from **(A)**. **(C)** Negatively stained EM of *M. sedula* CdvA double-helical polymers reconstituted *in vitro* that contained DNA. **(D,E)** Zooms of the emphasized areas indicated in panel **(C)**. scale bars 20 nm. **(F)** Negatively stained EM image of CdvBΔC polymers (Residues 1–167 -ΔMIM domain) from *M. sedula*. **(A,B)** are reproduced from Dobro et al. (2013) with permission. **(C–F)** Are reproduced from Moriscot et al. (2011) with permission.

As mentioned above, unlike the ESCRT-III proteins, CdvB does not bind membranes *in vitro*, likely because it lacks the CHMP3 basic patch that was implicated in its membrane binding (Samson et al., 2011). In contrast, CdvA does bind the archaeal tetraether polar lipid fraction E (PLFE) membranes. Cryo-electron microscopy has shown that CdvA polymerizes on the outside surface of PLFE liposomes and forms long polymers with a typical spacing of about 8 nm (see Figures 7A,B; Dobro et al., 2013). Furthermore, using the *S. acidocaldarius* CdvA-CbvB system, it was shown that CdvA could recruit CdvB to the membrane, and transform, in an CdvC-independent manner, small unilamellar PLFE vesicles into an extensive network of connected membrane tubes with a typical diameter of 10–20 nm (Samson et al., 2011). This might suggest that CdvB, like ESCRT-III complex, can directly cut narrow membrane necks. However, a note of caution should be added. Such a tubulation effect does not resemble the ordered bud-like structures that are formed when Vps20:Snf7:Vps24:Vps2 were incubated with small unilamellar vesicles (see Figure 6 in Saksena et al., 2009), and neither is it reminiscent of the intralumenal vesicles that were obtained by ESCRT-III from giant unilamellar vesicles (Wollert et al., 2009; Wollert and Hurley, 2010). In fact, it is not surprising that a membrane-associated dynamic polymer can tubulate membranes. Thus, the physiological relevance of this observation is not clear.

Another interesting feature of the CdvA filaments is their interaction with DNA. When *M. sedula* CdvA was purified from *E. coli*, it formed extended double helical filaments that copolymerized with the host DNA (see Figures 7C–E). It was impossible to remove the DNA from the filaments (Moriscot et al., 2011). Treatment of the CdvA filaments with DNAase resulted in disassembly of a large fraction of the filaments. This is probably a manifestation of the affinity of CdvA to the chromosome and may hint to CdvA participation in the chromosome segregation processes or suggest an inverse nucleoid-occlusion type localization mechanism for the archaeal divisome. Supplementing the CdvA filaments with CdvB did not cause any noticeable structural changes in the *M. sedula* CdvA filaments, which suggests a rather rigid structure for the CdvA filaments.

In contrast to CdvA, full-length *M. sedula* CdvB did not form *in vitro* polymers. Only a truncated version of *M. sedula* CdvB (CdvBΔC, residues 1–167), which includes only the ESCRT-III core domain, polymerized into extended filamentous structures *in vitro* (see Figure 7F). However, these unordered CdvB polymeric structures do not resemble the structurally defined reconstituted spirals or tubes that were found in ESCRT-III assays. At best, *M. sedula* CdvBΔ*C* polymers vaguely resemble the Vps24 reconstitution linear polymers (Ghazi-Tabatabai et al., 2008) or polymers of Snf7 mutants that are impaired in their function (Henne et al., 2012; Shen et al., 2014). Importantly, however, sucrose gradient centrifugation showed that, in spite of the proximity of the wH domain and the MIM2 motif, *the M. sedula* CdvB interacts in a non-mutually exclusive manner with both CdvA and CdvC (Moriscot et al., 2011), which supports the notion that the three proteins act synergistically.

Long and linear polymers of Cdv proteins were also detected when one of the Thaumarchaeota *Nitrosopumilus Maritimus* CdvB paralogs was expressed in yeast or mammalian cells (Ng et al., 2013). In that case, the polymers were dynamic, as judged by fluorescence recovery after photobleaching experiments. Similar to the *in vitro* CdvB polymers reconstitution, *N. Maritimus* CdvB paralog polymers needed only the core domain (residues 1–192) to polymerize. No other *N. Maritimus* CdvB paralogs formed polymers in yeast, leaving open questions regarding the operational state of the *N. Maritimus* CdvB paralogs *in vivo*.

## The ESCRT and Cdv machineries in cytokinesis

### The ESCRT-III in cell division

**Figure 8:**
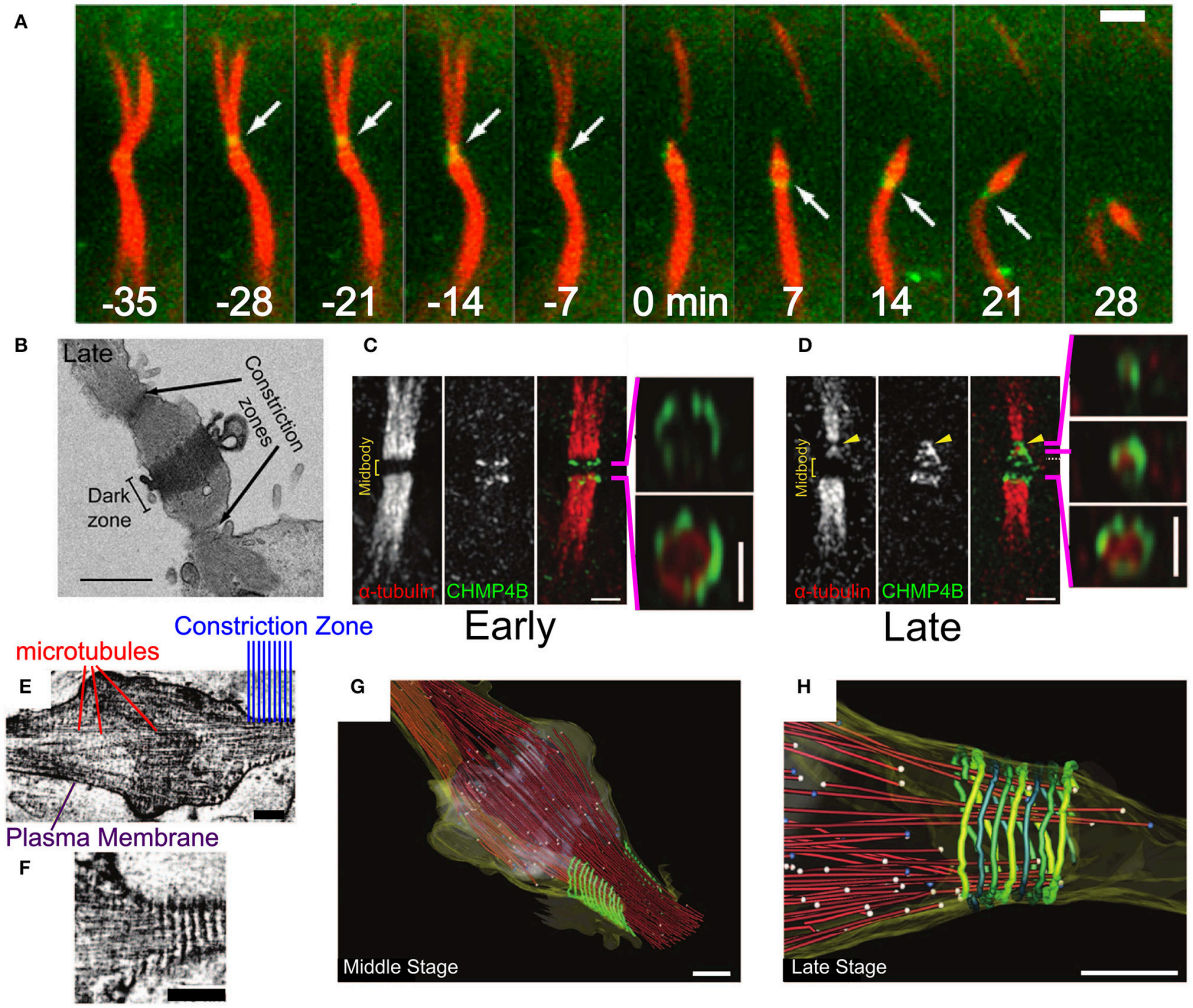
Abscission in eukaryotic cells during cytokinesis. **(A)** Abscission process of the intracellular bridge in Madin-Darby Canine Kidney (MDCK) Epithelial cells. CHMP4B in green and tubulin in red. White arrow—abscission site. Scale bar 2 μm. **(B)** Structure of the intracellular bridge in MDCK cells. Arrows indicate the future abscission sites. Scale bar 2 μm. **(C)** Early localization of GFP-CHMP4B (green) and mRFP α-tubulin to the division site of a HeLa cell. Panels on the right are 3D structured illumination (SIM) reconstructions of CHMP4B rings. Scale bar 1 μm. **(D)** Same as **(C)** at the late stage of abscission. Panels on the right are 3D SIM reconstruction of ESCRT-III cone. **(E,F)** Cryo-EM images of the Hela cell intracellular bridge at the middle stage of abscission. Scale bars—200 nm. **(E)** Shows the overall structure while **(F)** shows the cortical helical filaments. **(G–H)** Cryo-EM 3D reconstruction of a constriction zone. Red—microtubules, yellow—plasma membrane, green - 17 nm cortical filaments. **(G,H)** Shows the Middle and Late abscission stages, respectively. Scale bars—200 nm. **(A,B)** Is reproduced from Elia et al. (2011) with permission. **(C–H)** are reproduced from Guizetti et al. (2011) with permission.

The last stage of Metazoan cytokinesis involves the formation of a microtubules-rich intracellular bridge with a width of about 1 μm (see Figures 1, 8A,B). At the intracellular bridge middle, a protein-rich structure that is called the mid body (or Flemming body), is located. During late mitosis, Cep55 recruits ALIX and the ESCRT-I protein TSG101 to the mid body (Carlton and Martin-Serrano, 2007). These two factors recruit CHMP4 and initiate its polymerization (Morita et al., 2007; Carlton et al., 2008; Lee et al., 2008). Multiple experiments have shown that ESCRT-III is essential for cell separation in Metazoa (Elia et al., 2011; Guizetti et al., 2011; Lafaurie-Janvore et al., 2013). Here, we briefly describe its role in abscission. For a more detailed recent review on ESCRT in cytokinesis, see Stoten and Carlton (2017).

Initially, CHMP4 assembles in two parallel bands at the periphery arms of the Flemming body (see Figure 8C; Guizetti et al., 2011). Approximately 20–40 min later, ESCRT-III shuttles about 1 μm away from the mid body toward the abscission site that is located between the mid body and the cell (see Figures 8A,B) (Elia et al., 2011). There, it forms a cone-like structure that ends at the abscission site (see Figure 8D). Electron (Guizetti et al., 2011) and soft X-ray (Sherman et al., 2016) microscopy studies have detected a set of cortical filaments, each with a width of 17 nm, that are organized as a cone-like structure, similarly to the ESCRT-III complex (see Figures 8E–G). Just before abscission occurs, the ends of these cortical filaments structure correlate with the location of microtubules ends and with the abscission site (see Figure 8H). Although the cortical filaments are quite spaced and have a diameter of about 200 nm at their pointed end (about 2 times larger than the membrane neck at virus escape sites or the MVB buds), it is common to identify them with the ESCRT-III filaments. Thus, it was suggested that ESCRT-III is responsible for cell separation by remodeling the membrane at the abscission site. It is interesting to note that a cell-fate program determines whether abscission will also occur on the opposite side of the Flemming body, to leave an orphan mid body, or whether, alternatively, the mid body will be absorbed into the daughter cell cytoplasm (Chen et al., 2013).

Similar to other functions of the ESCRT machinery, also in cytokinesis, the ESCRT-III complex works concomitantly with VPS4. However, unlike in HIV-1 release or MVB formation, recruitment of VPS4A and VPS4B to the division site depends on IST1 (*K*_*d*_ ≈ 1 μM between IST1 and the Vps4 MIT domain; Agromayor et al., 2009; Bajorek et al., 2009a). Since the yeast homolog of CHMP1 (DId2) is responsible for the recruitment of Ist1 to the ESCRT-III complex at the MVB sites (Dimaano et al., 2008; Tan et al., 2015), it seems that IST1 is recruited to the ESCRT-III filaments at the abscission site by CHMP1. In addition, CHMP1A can bind VPS4 directly through its MIM1 motif with a *K*_*d*_ ≈ 4.5 μM, and recruit it to the ESCRT-III filament (Stuchell-Brereton et al., 2007). These data suggest that, at least for cytokinesis, the regulation of the VPS4 activity is mediated by high-affinity interactions in solution. After its recruitment to the division site, VPS4 can bind the lower affinity MIM1 of CHMP2B (Obita et al., 2007) or the MIM2 of CHMP6 (*K*_*d*_ ≈ 30 μM) (Kieffer et al., 2008), or even bind to CHMP4 (*K*_*d*_ > 100μM).

It should be noted that membrane deformation cannot be achieved without the destabilization of the intracellular bridge cytoskeleton components. Therefore, before abscission, spastin, a microtubule-severing protein, is recruited to the intracellular bridge by an interaction between its MIT domain and CHMP1B (Yang et al., 2008; Connell et al., 2009). Similarly, the recruitment of actin depolymerization and oxidation proteins was implicated for the completion of cytokinesis (Schiel et al., 2012; Frémont et al., 2017). These data show the differential mechanical requirements for the deformation of a bare membrane relative to those of the plasma membrane that is protected by a cytoskeleton. Interestingly, Metazoan cells apply an additional level of regulation to prevent premature abscission and persistent chromosomal bridges severing (the “NoCut” checkpoint; Carlton et al., 2012). Differential recruitment and phosphorylation of CHMP4 paralogs play a pivotal part in this checkpoint (Capalbo et al., 2012; Carlton et al., 2012). In particular, CHMP4C dephosphorylation and translocation from the center of the mid body to its periphery, after the “NoCut” checkpoint was resolved, is responsible for the initiation of mature ESCRT-III polymers, thus assuring cytokinesis completion (Capalbo et al., 2016).

Recently, it was found that during the second phase of the CHMP4B recruitment to the mid body, all the core components of the ESCRT-III machinery (CHMP4B, CHMP2B, CHMP3) showed an identical bipartite-population kinetics (Mierzwa et al., 2017). The first, a fast population has a residence time of about 20 s and is constantly exchanged with cytoplasmic proteins. The second, a slow population is exchanged only at the 10 min timescale. VPS4 showed a similar accumulation kinetics at the division site. Thus, the ESCRT-III-VSP4 machinery is highly dynamic during abscission. As discussed below, this might have implications on its membrane deformation mechanism. It is interesting that other components of the ESCRT-I/II complexes (besides TSG101 and ALIX) also play an important role in the recruitment of ESCRT-III components to the intracellular bridge and their dynamical shuttling away from the Flemming body (Christ et al., 2016). In particular, it was suggested that ESCRT-II, together with CHMP6, bridge the gap between the outer arms of the mid body and the abscission site, thus assuring the correct final positioning of the CHMP4B-CHMP2-CHMP3 polymers (Goliand et al., 2014).

Importantly, although the ESCRT system, most likely, directly remodels the membrane during abscission in Metazoan cells, this is not a universal modus operandi. For example, in budding yeast, ESCRT plays only a minor role in cell division. Indeed, it was suggested that ESCRT's primary function in *S. cerevisiae* division is to mediate the turnover of cell-division proteins from the plasma membrane (McMurray et al., 2011), Similarly, in *S. pombe*, the main role of ESCRT was ascribed to the control of membrane trafficking during cytokinesis (Bhutta et al., 2014). Finally, in Arabidopsis, elch, a homolog of the ESCRT-I TSG101 protein, functions in cytokinesis (Spitzer et al., 2006). Yet again, in that case, its main role is related to the regulation of the microtubule cytoskeleton. In the context of cytokinesis, the main relevant difference between plants, fungi, and Metazoa may be the existence of a cell wall in the former cases. Accordingly, for understanding the relationship between the ESCRT and the Cdv systems, it is interesting to check whether the ESCRT system remodels the membrane directly during cytokinesis in other lower cell-walled eukaryotes.

### The Cdv system in division

First experimental evidence that linked Cdv proteins to cell-cycle regulated processes and in particular to cell division came from UV radiation studies. Exposure of *S. solfataricus* cells to UV radiation caused a severe down-regulation of the transcription levels of all cdv genes (Fröls et al., 2007). Similarly, for *S. acidocaldarius*, the main locus cdv genes, as well as cdvB2 and cdvB3, were down-regulated (Götz et al., 2007). In accordance with these observations, in *S. acidocaldarius*, all cdv genes are cell-cycle regulated, with cdvA, cdvB, and cdvB2 showing the largest increase (about 3.5-fold) before entering the division phase (Lindås et al., 2008; Samson et al., 2008).

**Figure 9:**
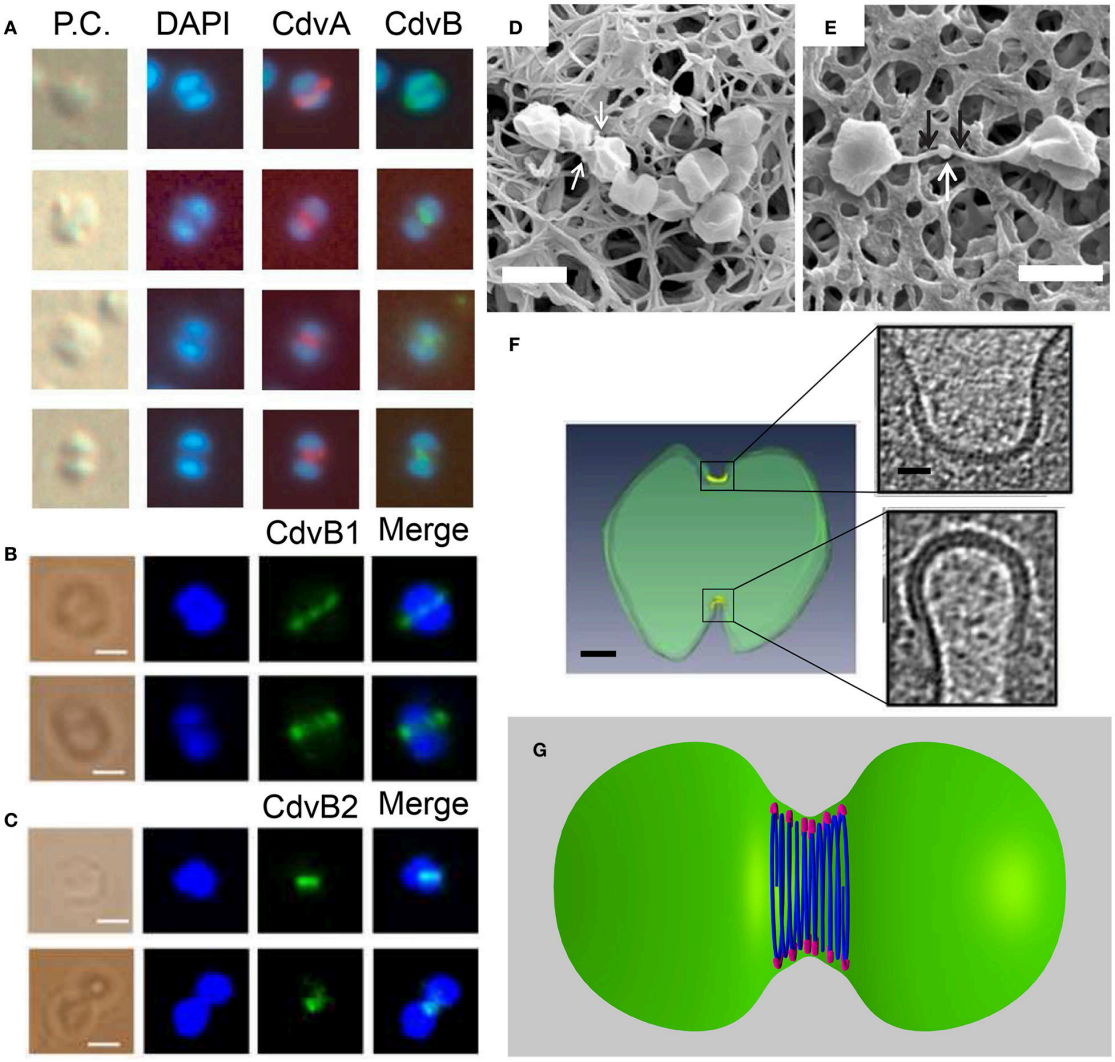
The Cdv system in cytokinesis in Crenarchaeota. Left column: *In situ* immunofluorescence microscopy of Cdv proteins. **(A)** Intermediate steps during *S. acidocaldarius* cytokinesis showing localization of CdvA and CdvB bands that shrink in concomitant with the septum formation. P.C. denotes phase contrast. **(B,C)** Localization of *S. islandicus* CbvB1 **(B)** and CdvB2 **(C)** to the division site. The localization is visualized before chromosomes segregated (upper row in each panel) and during cytokinesis after chromosome segregation (lower raw in each panel). Scale bars 1 μm. **(D)** Mutant *S. islandicus* cells expressing a reduced level of CdvB1 are locked in a “chain-like” phenotype and cannot separate. **(E)** Mutant *S. islandicus* cells over-expressing CdvB2 form a “mid-body” like phenotype. Scale bars for **(D,E)** 2 μm. **(F)** Cryo-EM segmented image of a dividing *S. acidocaldarius* cell. Cell membrane is denoted in green and the thick protein belt at the cleavage furrow is denoted in yellow. Insets - zoom in of the two sides of the cleavage furrow. Scale bars - 150 nm for the whole cell, 40 nm for the insets. **(G)** “Hourglass” model of the Cdv system during cytokinesis in dividing *S. acidocaldarius* cell, showing CdvB polymers (blue) that are connected to the membrane via CdvA (purple). For clarity, CdvA is shown only at the top and bottom of the cell. In reality, it is located along the whole perimeter of the cell. **(A)** Is reproduced from Lindås et al. (2008) with permission, Copyright (2088) National Academy of Sciences. **(B–E)** Are reproduced from Liu et al. (2017) with permission. **(F)** Is reproduced from Dobro et al. (2013) with permission.

The most direct evidence that connects the Cdv system to cell division comes from immunofluorescence microscopy experiments. These studies revealed the localization of CdvA and CdvB to the division site between segregated chromosomes, and specifically the formation of a band that shrinks concomitant with the septum formation (see Figure 9A). CdvC localizes to the division site as well, but in some cases, a diffusive pattern without a clear-cut localization is observed. Treatment of *S. acidocaldarius* cells with antibiotics that arrest the cell cycle prior to division resulted in the abolishment of the Cdv bands (Lindås et al., 2008). Accordingly, treatment of cells with tunicamycin, an antibiotic that causes cell-cycle arrest at the division phase, resulted in a 3-fold increase in the abundance of Cdv mid-cell bands. Overall, the combined data clearly indicate that the Cdv system is a major player in Sulfolobales cell division.

A detailed analysis of the main Cdv locus showed that it is composed of two transcription units, one for CdvA, and the second one for CdvB and CdvC (see Figure 2B). Transcription-level measurements showed that the up-regulation of cdvA preceded that of all four cdvB paralogs by ~30 min (Samson et al., 2011). This suggests that CdvA mediates the division apparatus localization and recruits the downstream CdvB proteins. In support of this hypothesis, immunofluorescence microscopy showed a large number of CdvA bands that are localized outside the mid-cell, perpendicular to the division site or even across non-segregated chromosomes (Samson et al., 2011). In contrast, the CdvB band was detected only at mid-cell and rarely over non-segregated chromosomes (Lindås et al., 2008). Together, the data suggest that CdvA transduces the information regarding chromosome segregation to the assembly of the division apparatus.

Mutational and over-expression assays further implicated a central role for the Cdv system in Crenarchaeota cell division. Expression of a dominant-negative ATP-hydrolysis-deficient CdvC^*E*260*Q*^, resulted in the formation of large cells that contain less than one (indicating apoptosis) or more than two (indicating a cell-cycle arrest) genome equivalents in the mutant strain (Samson et al., 2008). Similarly, over-expression of the CdvB wH domain resulted in large cells that were devoid of chromosomes (Samson et al., 2011). An additional study showed that deletion of either *S. acidocaldarius* CdvB1 or CdvB2 resulted in the formation of large cells with an increased amount of DNA (Yang and Driessen, 2014). This aberrant phenotype was accompanied by slower growth in liquid and the formation of smaller colonies on agar plates. Yet, these mutants were viable. This might indicate that CdvB1 and CdvB2 can functionally substitute for each other, albeit with some deficiency.

By contrast, deletion of CdvB3 resulted in a more severe phenotype. The growth rate in liquid was very slow, colonies did not grow on agar, and the cells were much larger in comparison to the ΔCdvB1 and the ΔCdvB2 mutants. In addition, the localization pattern of CdvA was aberrant, and many CdvA bands were localized at the edge of the cell, adjacent to the edge of the chromosome. Thus, CdvB3 plays an important but non-essential role in cell division. It will be interesting to check if the Ist1 homology that we identified contributes to this phenotype through interaction with CdvB1.

In contrast to *S. acidocaldarius*, in *S. islandicus* the function of the CdvB paralogs in cytokinesis appears to be reversed (Liu et al., 2017). Here it was found that while CdvB3 is dispensable for cell division and mainly acts in viral release, no viable ΔCdvB1 or ΔCdvB2 mutants could be constructed. Immunofluorescence microscopy detected a band of CdvB1 and CdvB2 at the division site, between segregated chromosomes or even before chromosome segregation (see Figures 9B,C). A mutant strain with a negligible expression of CdvB1 showed an aberrant phenotype that occurred in about 35% of the cases (see Figure 9D) where cells could not complete their division and stayed connected as a non-separable chain of cells. Over-expression of CdvB1-ΔC (which lacks the truncated MIM2 motif), also resulted in the formation of chains of cells. In contrast, over-expression of CdvB-ΔC arrested the cells in a peanut-like shape in about 33% of the cases. Thus, these data suggest that, while CdvB is mainly important for the execution of the early stage of division, *S. islandicus* CdvB1 acts at a later stage that is related to furrow maturation and final cell separation. Strikingly, over-expression of CdvB2-ΔC resulted in a phenotype where the two daughter *S. islandicus* cells stayed connected by a long (>2 μm) and narrow (~100 nm) membrane tube with a blob at its center (see Figure 9E), a phenotype that clearly is reminiscent of the intracellular bridge and the mid-body of Metazoan cells. Both CdvB1 and CdvB2 localized to these mid-body-like structures, suggesting a role for these proteins (especially for CdvB2) in abscission. It is interesting to ask about the source of these different behaviors of CdvB1/2 mutants in *S. acidocaldarius* and *S. islandicus*. One possible explanation might be related to their interaction with CdvC which was not detected for *S. acidocaldarius* but was detected for *S. islandicus*. In particular, it should be interesting to check if these differences result from the Vta1-like homology in *S. islandicus* that we identified.

The overall picture that emerges from these biochemistry and cell-biology assays is that the Cdv system is a key player in cell division. Yet, a clear understanding of its biophysical mechanics is still missing. In *S. acidocaldarius*, Z-stack reconstruction of the CdvA band showed that it forms both open and closed rings (Samson et al., 2011). These data might suggest a similar biophysical mechanism as that of the bacterial FtsZ, which also forms non-continuous rings (Fu et al., 2010; Strauss et al., 2012; Holden et al., 2014). Cryo-EM tomography of dividing *S. acidocaldarius* cells, however, has detected a full-circle proteinaceous band with a radial thickness of ~3.5 nm that was separated from the membrane by a distance of ~6 nm in all cells, irrespective of the furrow ingression stage (see Figure 9F; Dobro et al., 2013). As cell division progressed, the width of the belt on the cell surface increased from 150 to 400 nm, while its thickness remained constant. As a result of the progressive septum formation and the increase of the belt surface area, the curvature of the belt also became larger, likely due to an active process of increasing Cdv protein mass at the division site as cytokinesis progresses. Interestingly, in all cases, the division furrow at one side of the cell was more advanced and differently shaped than that on the other side. In fact, asymmetric division is also observed in many other bacterial and archaeal species, including those that use FtsZ (Yao et al., 2017). In addition, the surface layer (the proteinaceous cell-wall layer of archaea) was often incomplete, and many budding vesicles were detected at the division site. These data suggest a dynamic, asymmetric and cell-wall- and plasma-membrane-coupled division process. It was interpreted as representing a dense “hourglass” belt of CdvB that is coupled to the membrane through a sparse CdvA layer (see Figure 9G). According to this model, the increase in belt width represents an addition of CdvB coils, and coils at the middle of the furrow shrink faster than those at the belts side. In this framework, the reduction in diameter of the central CdvB coils is the driving force for cytokinesis. Yet, more data are needed before the full biophysical mechanism of Sulfolobales division can be inferred.

It is interesting to note that the localization picture of Cdv proteins was different for the rod-shaped Thaumarchaeon *N. maritimus* (Pelve et al., 2011). Here, CdvA and CdvC localized to the middle of the cell, in most cases correlated with chromosome segregation, but none of the CdvB paralogs showed the formation of a mid-cell band. The strength of the fluorescence signal of the CdvB1/2 paralogs (Nmar_0816 and Nmar_029) was correlated with the formation of CdvA-CdvC mid-cell localized band, but both CdvB paralogs showed a diffusive fluorescence pattern along the whole cell length and did not localize to a specific location. In fact, such a diffusive fluorescence signal was also observed for the *N. maritimus* FtsZ homolog, which lacks the conserved signature sequence for GTP binding that is crucial for FtsZ polymerization (Busiek and Margolin, 2011).

As is discussed below, these data might suggest that the division mechanism in some TACK organisms is in fact not directly related to the ESCRT-III membrane remodeling mechanism.

## The mechanism of the Cdv and ESCRT machineries

### Models of the ESCRT-III membrane remodeling

**Figure 10:**
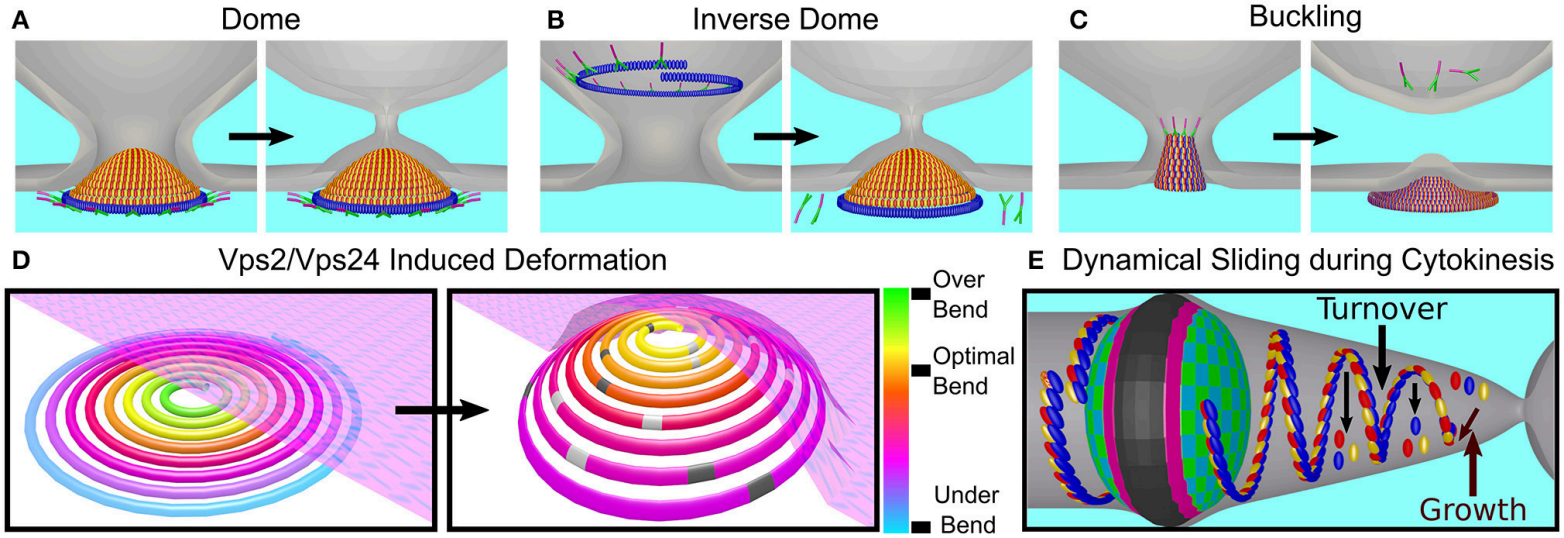
Models of bud neck abscission by ESCRT-III. The function of ESCRT-I-II (green and pink) and ESCRT-III (red, Vps2; yellow, Vps24; and blue, Snf7) are shown for different models for the mechanisms of membrane remodeling by ESCRT proteins. **(A)** Dome model—ESCRT-I/II complex initiates the formation of an Vps2/24 dome from the cytoplasm side. The membrane is remodeled as results of its wrapping up tightly around the dome. **(B)** Reverse dome model - same as the dome model only that the ESCRT-III structure is initiated from the bud lumen. The bud grows but then turn over to end up in a configuration similar to the one in the dome model. The driving force for membrane remodeling is again the tight binding to the ESCRT-III dome. **(C)** Stress-induced buckling model—polymerization of ESCRT-III polymers results in the accumulation of stress due to the deviation from the preferred curvature. As a result, the ESCRT-III structure is transformed from a flat 2D one to a 3D spiral. Reverse buckling than causes the abscission of the membrane. **(D)** Membrane-curvature deformation upon Vps2/Vps24 (light and dark gray) binding to an Snf7 filament. According to this model, the driving force for the deformation of the ESCRT-III structure is the binding of Vps2/Vps24 for the Snf7 polymer. The sporadic binding creates a locally preferred curvature that is different from that of the Snf7 polymer. This promotes the buckling of the ESCRT-III structure. Since the membrane binds the ESCRT-III structure, it is remodeled by its buckling. **(E)** Dynamical polymerization of ESCRT-III filaments leading to membrane ingression - In this model, the ESCRT-III structure is not static, but dynamic. Polymerization and depolymerization from the end of the ESCRT-III structure, as well as from its middle, continually contribute to monomers turnover and global structure deformation that results in the membrane remodeling.

Different classes of theoretical models have attempted to explain membrane deformation by the ESCRT-III complex based on the *in vivo* data and the *in vitro* reconstitution of ESCRT-III higher-order structures. The first of these are dome-like models. The direct dome model was inspired by the dome-like structures that are observed at the tip of the CHMP2A:CHMP3 tubes (see Figure 6C; Fabrikant et al., 2009). According to this model, Snf7/CHMP4 polymers encircle a membrane patch and recruit Vps2/CHMP2 and Vps24/CHMP3 to form a dome-like structure within a narrow membrane neck with a typical diameter of 50 nm at its base (see Figure 10A). The key ingredient of this model is that a high binding affinity between the charged lipids and dome proteins compensate for the bending energy that is involved in membrane deformation so that the membrane follows the dome shape. At the tip of the dome, spontaneous fission can then occur. Similar to the case of MVB formation, a dome-like structure was predicted to drive membrane abscission during cytokinesis (Elia et al., 2012). However, the ESCRT system cannot remodel the membrane next to the Flemming body due to its size and rigidity. Instead a breakage of the filaments and their sliding away from the Flemming body toward the abscission site is predicted, where mechanic and elastic considerations allow the functioning of the ESCRT dome.

For HIV-I release, the major viral Gag protein binds the cell membrane, produces the early viral bud, and recruits ALIX and TSG101 to initiate CHMP4 polymerization. Thus, according to the dome-like model, the dome would cut the membrane from the virus side. However, deep-etch EM microscopy showed that the ESCRT-III funnel starts at the narrow part of the bud neck and widens toward the cytoplasm (Cashikar et al., 2014). This finding is also consistent with the fact that ESCRT-III proteins are generally not found in viral bodies after their release. Thus, a variation of the dome model, namely the inverse dome model, was suggested (see Figure 10B). According to this model, ESCRT-III polymerization is initiated from the virion side and grows from a large radius to a smaller one. At a certain point, the direction of growth is inverted, and the dome ends up as in the original dome model (Schöneberg et al., 2017).

A second class of models is based on the mechanical properties of CHMP4/Snf7 spirals structures. As was discussed above, a 2D spiral polymerization results in stress accumulation (Shen et al., 2014; Chiaruttini et al., 2015), which can be relieved if the spirals buckle into the form of a tube (Lenz et al., 2009). Since the membrane follows the ESCRT-III shape due to binding interactions, a tubular-shaped membrane can thus be formed. This model, however, does not explain how ESCRT-III induces membrane fission. Hence, it was suggested that for membrane fission, buckling occurs in the opposite direction, that is, from a tubular to a flat shape (see Figure 10C; Carlson et al., 2015). However, the physical basis for this hypothetical reversed buckling is unclear.

A variation of the buckling model suggests that the unique preferred curvature of various ESCRT-III subunits is the key factor for membrane abscission (Chiaruttini and Roux, 2017). Based on *in vitro* observations, it was suggested that *in vivo*, CHMP2/Vps2 and CHMP3/Vps24 might induce a localized change in the CHMP4/snf7 spirals curvature, and hence buckling (see Figure 10D). Still, similar to the simple stress-induced buckling mechanisms, it is not clear what will be the driving force for membrane fission. One possibility is that differential removal of subunits with a certain preferred curvature by Vps4 may drive shape transformation of the ESCRT-III polymers. Such differential removal of CHMP proteins can occur, for example, as result of the fact that different ESCRT-III subunits have a different affinity for Vps4 (as mentioned above there exist two orders of magnitude difference between the affinities of CHMP2A and CHMP4 for Vps4).

Finally, a third class of ESCRT-III abscission model, based on recent *in vitro* measurements of a high turnover rate of ESCRT-III monomers and similar dynamics *in vivo*, suggests that ESCRT-III are “live” polymers that are constantly remodeled by Vps4 (see Figure 10E; Mierzwa et al., 2017). In this model, Vps4 may lead to growth of the polymer tip or to a differential removal of ESCRT-III subunits from the polymer core. Very recently, the dynamics of ESCRT proteins during MVB formation was measured using light-sheet microscopy (Adell et al., 2017), yielding also a high turnover rate and number fluctuations of ESCRT-III proteins that were coupled to Vps4 recruitment. The authors suggested that their data were inconsistent with existing ESCRT models. Instead, they suggested that ESCRT-III polymerizes and depolymerizes independently of Vps4. They further suggested that the main function of Vps4 is to bind together two or more ESCRT-III polymers, which can occur as result of the multiple binding sites for ESCRT-III that Vps4 possesses. This may result in condensation of the polymers network that, together with limited cargo space, results in membrane invagination, reduction of the bud neck size, and finally abscission. Yet, a detailed physical understanding of how this process, or alternative processes that depend on a high turnover rate of the ESCRT-III proteins, induce membrane remodeling and fission is still missing.

### Comparison of the Cdv and ESCRT-III systems

A breadth of experimental results has shown that the Cdv system is a key participant in cell division in Sulfolobales (and probably in Thermoproteales). Similar to the scenario in bacteria, there are two options for a mechanism that can underlie the division process. The first is that CdvB paralogs apply a force on the membrane leading to its deformation, similar to the ESCRT-III case. The second is that the Cdv system merely acts as a scaffold to coordinate coupling to cell wall deformation and local vesicle secretion/fusion. Here, we discuss these two possibilities.

The first point to consider in this context is the typical length scale over which the Cdv and ESCRT systems function. During cytokinesis, the ESCRT-III complex is initially assembled at the Flemming body that is about 1 μm in size. However, the ESCRT system cannot remodel the membrane at that site. Instead, it slides to the abscission site, which has a diameter of approximately 100 nm, similar to other ESCRT-III functioning sites (such as MVB or virus release buds). Thus, the ESCRT system acts only in the very last step of membrane abscission, while the diameter of the abscission site probably mainly depends on actin and tubulin remodeling. In contrast, the Cdv system acts throughout the whole phase of septum ingression, from its start where the diameter of the cell is about 1 μm to its end. Thus, it is not clear how the supposedly more primitive system (the Cdv system) can accommodate a more robust function over a broad span of membrane widths if the two systems share the same mechanism.

Another important point relates to the CdvB-CdvC affinity. For the ESCRT system, a span of interaction strengths exists between Vps4 and various ESCRT-III subunits. Some of these interactions are high-affinity, and they are necessary for the efficient recruitment of Vps4 from solution to the ESCRT-III complex (e.g., the IST1-VPS4 interactions in cytokinesis). However, for *S. acidocaldarius*, the MIM_*CdvB*_-CdvC affinity is only moderate. Indeed, CdvC acts as a hexamer, and thus the apparent affinity to the CdvB band should be higher. Still, as Animalia use the large span of affinities of ESCRT-III proteins to VPS4 so that abscission will be efficient, it is unclear how an efficient recruitment of CdvC from the cytoplasm is achieved in Crenarchaeota. If the recruitment efficiency of CdvC is much lower than in Metazoa, it may imply that the turnover rate of CdvB is lower. Thus, the recent suggestion that ESCRT-III can cut the membrane because of a high turnover of the subunits might not be relevant in archaea. One way to check this issue is to measure the ATPase-activity simulation of CdvC by CdvB.

An additional point to consider is the current lack of evidence for the formation of higher-order structures such as spirals, tubes or domes by CdvB paralogs *in vitro*. Of course, this can merely be an experimental issue that necessitates more efforts. Still, such evidence is awaiting, and this is especially important if ESCRT-III acts as a spiral spring. Since the sine qua non skeletal component of ESCRT-III spiral is Snf7, while CdvB is a homolog of the Vps2/Vps24/Did2 class, which shares a very low sequence homology with Snf7, it is a priori unclear if CdvB paralogs are geared to form spirals. Moreover, for a spiral-based mechanism to function, there must be an asymmetry between the base of the spiral to its buckled tip where abscission occurs. In the Cdv case, however, the band at the division site has a symmetric “hourglass” shape (Dobro et al., 2013), which probably precludes buckling-like mechanisms for the Cdv system.

A similar problem arises concerning the dome-based models. The dome model is based on the assumption that the membrane has a high affinity to the dome subunits (Fabrikant et al., 2009). These strong interactions compensate for the high energy penalty that is associated with bending the membrane. For the model to work, given reasonable affinity values, it is essential that every subunit will interact with the membrane. However, CdvB does not bind the membrane at all, and it is not clear, theoretically, if the sparse binding of CdvA to the membrane (Dobro et al., 2013) is sufficient to fulfill the model requirements. Of course, one may speculate that other CdvB paralogs may bind the membrane, but this remains to be shown.

Finally, there is a question regarding the actual orientation CdvB relative to the membrane. This problem stems from the fact that CdvA polymerized on the outside surface of liposomes (see Figure 7B; Dobro et al., 2013). Indeed, the curvature of these liposomes was not large, but if CdvA, which is supposed to act as the sole recruiter of CdvB, can bind the membrane from both sides, it raises questions regarding the orientation of CdvB relative to the membrane *in vivo*. Recall that for the ESCRT system, inversion of the membrane binding direction transferred the ESCRT-III proteins from an inverse-topology to a direct-topology membrane remodeling machinery (see Figures 3F, 6D for the CHMP1B-IST1 case). Similarly, for bacteria, inversion of the FtsZ membrane binding direction caused it to form bands on the outside surface of vesicles instead of the inside surface (Osawa and Erickson, 2011). Thus, for the development of a comprehensive model of the Cdv system, future experiments will have to clarify the orientation of CdvA relative to the membrane.

Irrespective of the previous arguments, the strongest argument against the Cdv and the ESCRT-III having the same biophysical mechanism is the existence of the proteinaceous Surface-layer (S-layer) in the first case. The Sulfolobales S-layer is a cell-wall-like layer, and the sole protector against the turgor pressure (Engelhardt, 2007; Albers and Meyer, 2011). For a thin porous S-layer to function as a mechanical protection against turgor, the crystalline distance between adjacent S-layer monomers should be small, on the order of a few nm (Engelhardt, 2016). Otherwise, the membrane will bulge out. For Sulfolobales division, it was reported that, surprisingly, the S-layer was often incomplete (Dobro et al., 2013). Large voids in the S-layer would undoubtedly result in bulging out of the membrane, suggesting the presence of another supportive layer that can replace the S-layer locally and temporarily. We speculate that one of the functions of the dark band that was detected at the *S. acidocaldarius* division site (Dobro et al., 2013) is to protect the cell during membrane ingression (see Figure 9F). This might explain why this band was highly dense, in comparison to the sparsely distributed 17 nm cortical eukaryotic filaments (see Figures 8G,H). As mentioned above, at the *S. acidocaldarius* division site, many budding vesicles were observed. Note that also in Fungi and plants, the participation of the ESCRT system in cell division is related to vesicles delivery. In addition, it was suggested that the function of the ESCRT system during the abscission phase of the *C. elegans* embryo first division is to facilitate membrane removal (König et al., 2017). Thus, if the Sulfolobales division apparatus is composed of several proteins in addition to the Cdv system, the function of the Cdv system may be to deal with surplus membrane invagination, while other proteins may provide the mechanical support. Concomitantly, during cytokinesis, the S-layer should first be removed and then rebuilt at the septum site to reshaping the cell.

In light of this discussion we prefer the explanation that, in Crenarchaeota, the primary function of the Cdv system is related to vesiculation of the plasma membrane while other cofactors establish an inner protection layer against turgor. A comparison to the bacterial FtsZ may be fitting here: For many years, it was assumed that the primary function of FtsZ is to apply force on the membrane. However, it was recently shown that a major role of FtsZ is to coordinate the synthesis of cell wall material (Bisson-Filho et al., 2017; Yang et al., 2017). We also would like to suggest that CdvA's role in cytokinesis is more important than merely to recruit CdvB to the membrane. It will be interesting to check these hypotheses experimentally.

While above we discussed the mechanistic aspects of the Cdv and ESCRT-III systems, it also is of interest to discuss their evolutionary relationship. There are two possible evolutionary scenarios. According to the first, the Cdv system was a reversed-topology membrane-remodeling machinery that continued to develop in eukaryotes but sometimes lost its cytokinetic functionality (e.g., for yeast and plants). According to the second, the Cdv system developed its cytokinesis function independently in Crenarchaeota and Animalia and that this pattern represents a form of functional convergent evolution, where homologous protein systems participate in a similar biological pathway, but their role in this pathway is different due to a lack of evolutionary continuity. We argue that if indeed our mechanistic hypothesis is correct, and the Cdv system does not remodel the membrane directly, the second evolutionary scenario is more likely. One way to further study the evolutionary relationship of the ESCRT/Cdv systems is to study their functional mechanics in protists and the archaeal Asgard phylum, to see if they function similarly as in animals.

## Conclusions and outlook

During the last two decades, the eukaryotic ESCRT field was established and matured. We hope that, similarly, the next decade will considerably expand our understanding of the archaeal Cdv system. We envision that structural biology studies will continue to shed light on the function of Cdv proteins. In particular, high-resolution structures of CdvA and the different CdvB paralogs would be beneficial to assess their role in cytokinesis. Similarly, high-resolution structure of CdvC in its ATP-bound form should show whether it forms a helical hexamer, like Vps4, or whether it forms a hexameric ring also in its active ATP-bound state.

*In vivo* studies, and especially the application of advanced light microscopy tools to study the Cdv system in real time, bear great potential to expand our understanding of the Cdv system. In particular, such studies should be able to disentangle the exact relations between chromosome segregation and CdvA localization. They should also allow to identify the possible existence of regulatory mechanisms similar to the “NoCut” check-point, which assures DNA clearance from the division site before cytokinesis. In this context, it will also be interesting to study the possible interaction of CdvA and the DNA and, if found *in vivo*, to understand its possible physiological consequences. A particular emphasis should be directed to study the exact function of CdvB paralogs in different Crenarchaeota species, and to decipher the coupling to CdvC and the S-layer synthesis machinery. The study of mutations of Cdv proteins will undoubtedly contribute significantly in future work on the system, and will likely serve as a valuable source for deciphering its mechanism of action. More advanced *in vitro* studies that will reconstitute the Cdv system in vesicles and synthetic cells (Caspi and Dekker, 2014; Härtel and Schwille, 2014) or on supported lipid membranes will add invaluable information regarding the different higher-order structures that the CdvA/CdvB paraloges system can form. In particular, such studies will be able to show if CdvB paralogs can form spirals or domes like the ESCRT-III subunits.

Altogether, we are optimistic that future experiments will clarify the biophysical mechanism as well as the evolutionary role of the Cdv system. We hope that this review has been of help to clarify some important points and expand the discussion regarding the archaeal Cdv system in cytokinesis. We also hope that it will attract new scientists to study archaeal division, which, in every sense, constitutes a fascinating topic to study in the context of the great variety of cell-division modes that exist in nature.

## Author contributions

All authors listed have made a substantial, direct and intellectual contribution to the work, and approved it for publication.

### Conflict of interest statement

The authors declare that the research was conducted in the absence of any commercial or financial relationships that could be construed as a potential conflict of interest.
