# Supplementary material for: Dividing the Archaeal Way: The Ancient Cdv Cell-Division Machinery
Source: Front Microbiol. 2018 Mar 2;9:174. doi: 10.3389/fmicb.2018.00174 (PMC5840170; doi:10.3389/fmicb.2018.00174)
Supplement: Supplementary file 1 [file DataSheet1.pdf]

Dividing the archaeal way: the ancient Cdv cell-division machinery

Yaron Caspi* and Cees Dekker

Department of Bionanoscience, Kavli Institute of Nanoscience, Delft University of Technology, Van der Maasweg 9, 2629 HZ Delft, The Netherlands

*Current Address: Brain Center Rudolf Magnus, University Medical Center Utrecht, Universiteitsweg 100, 3584 CG Utrecht The Netherlands

Supporting Information

Methods

Blast results were obtained using the NIH NCBI protein BLASTP 2.6.1 web portal. The quarries for proteins was based on the *S. acidocaldarius* Cdv proteins sequence. Subject sequences were derived based on the genome sequence of the specified archaea species. For general blast search for CdvB/CdvB1/CdvB3, we used the DELTA-BLAST (Domain Enhanced Lookup Time Accelerated BLAST) algorithm. For the rest of the cases, we used the blastp (protein-protein BLAST) algorithm. For direct homology values extraction, we used a direct sequence-sequence alignment blast based on the identified genes and corrected the results based on Ref. [Spang *et al.*, 2015] and Ref. [Makarova *et al.*, 2010]. Blast of ESCRT-I and ESCRT-II was done against a quarry of the *S. cerevisiae* Vps25 and Vps28 proteins. The Vst1 quarry was based on the sequence of the protein data bank structure #2LUH [Yang *et al.*, 2012]. Protein tertiary structure for *S. acidocaldarius* CdvB (Saci_1373) was obtained using the Phyre2 web application (http://www.sbg.bio.ic.ac.uk). Protein structure representation was constructed using the UCSF Chimera program (RRID:SCR_004097). Structure alignment was obtained using Chimera alignment tool, using the command: "mmaker #0 #1 pair bb alg nw matrix BLOSUM-62 showAlignment true iter 10". The Cdv protein domain search was based on the results of the NCBI protein analysis and/or the domain analysis web servers InterPro (https://www.ebi.ac.uk/interpro) and GenomeNet motif search (http://www.genome.jp/tools/motif/). Secondary structure prediction for *S. cerevisiae* Vps2 and *S. acidocaldarius* CdvB was done using the predictprotein.org web server (https://www.predictprotein.org). The phylogenetic tree was build using multiple-alignment by Muscle followed by tree construction using PHYLIP Neighbor-joining as part of the Unipro Ugene program suite (RRID:SCR_005579). All figures were prepared using Inkskape (RRID:SCR_014479) and Blender (RRID:SCR_008606). The hydropathy index calculation was done using the Innovagen peptide calculator, (www.pepcalc.com) which is based on the Hopp and Woods method.

The Cdv system in exovesicles and viruses

Exovesicles are secreted membrane vesicles that are released from the plasma membrane and participate in various biological processes. LC-MS proteomics analysis of the exovesicles from *S. acidocaldarius* identified the presence of CdvB1, CdvB2, and CdvC in the secreted vesicles [Ellen *et al.*, 2009]. Similarly, for *S. solfataricus*, CdvB1/2/3 and CdvC were identified in the exovesicle content. Indeed, both *S. acidocaldarius* CdvB1 and CdvB2, as well as *S. solfataricus* CdvB2 and CdvC were among the few proteins that could be clearly detected on an SDS-PAGE gel analysis of the exovesicles content. Since the detection sensitivity of gels is quite limited, this suggests that these Cdv proteins are major components of the exovesicles content. In addition, immunogold labeling in *S. solfataricus* identified CdvC as located mainly on the plasma membrane of non-dividing cells [Ellen *et al.*, 2009], consistent with its role in exovesicles formation. Together, these data suggest that the Cdv system plays a major role in Sulfolobales exovesicles formation, and that CdvB1 and CdvB2 are the main players in this process. However, it is yet unclear how CdvC is recruited to the exovesicles release sites in *S. acidocaldarius*, since both CdvB1 and CdvB2 have a very low binding affinity to CdvC. In addition, these data stand in contrast to the mechanisms of action of the eukaryotic ESCRT-III system that, in most of the cases, leaves behind little non-recycled traces in the lumen of exovesicles [Olver & Vidal, 2007].

A function for the Cdv system was also implicated in virus release. During the release of *Solfolobus* Turreted icosahedral virus (STIV) from *S. solfataricus,* a pyramid-like structure sprouts from the plasma membrane while breaking up the rigid cell S-layer [Snyder & Young, 2011]. Transcription analysis of cells after STIV infection revealed that the cdvA, cdvB, cdvC, and cdvB2 genes were upregulated [Ortmann *et al.*, 2008]. Similarly, proteomics analysis identified upregulation of CdvA, CdvB1, and CdvB2 [Maaty *et al.*, 2012]. This is especially important since only ten host proteins were identified by the proteomics analysis as having differential expression levels. In addition, CdvB3 was one of the two host proteins that were purified with the virions [Maaty *et al.*, 2006]. Importantly, yeast two-hybrid system revealed direct interactions between CdvB3 and one of the major capsid proteins as well as between CdvB and the STIV C92 protein, which solely forms pyramid-like structures when expressed in *E. coli* [Snyder *et al.*, 2013]. Final support for the close connection between the Cdv system and the STIV virus release mechanism comes from transmission electron microscopy immunogold labeling of CdvC and CdvB. Both proteins were detected in the vicinity of the sprouting pyramid structure. However, while CdvB was detected at the virion base as well as at its apex, CdvC was mainly detected outside of the virion but next to its apex [Snyder *et al.*, 2013]. Thus, STIV probably makes use of the Cdv system for its release, but the exact mechanism is still unknown. Recalling that the ESCRT system leaves little traces in the virion lumen [Olver & Vidal, 2007], these data might suggest, similar to the exovesicles case, a somewhat different function during viral release in eukaryotes and archaea. In particular, in this context, it is important to remember that in eukaryotes, virus release is mediated through abscission of the narrow membrane neck of the virus envelope. In the STIV case, the base of the pyramid, which is the last to detach from the plasma membrane is the widest part of the virion particle. Hence, in the context of viral release, the ESCRT and the Cdv system act in a different geometrical situation.

In fact, recently it was shown that the deletion of the *S. islandicus* CdvB3 (but not of the truncated versions of CdvB1 or CdvB2) blocked the formation of viral particles in cells that were infected by the Sulfolobus tengchongensis spindle-shaped virus 2 (STSV2) [Liu *et al.*, 2017]. In addition, over-expression of CdvB3 (but not the other CdvB paralogues) produced buds from the plasma membrane, and both CdvB1, CdvB2, and CdvB3 were recruited to the budding sites.

Together, these data point to the important role of the Cdv system in the life cycle of some Crenarchaeota viruses and suggest that, similar to eukaryotes, also among the Sulfolobales, viruses can hijack the Cdv system. Concordantly, in the Acidianus Tailed Spindle Virus, an integral viral protein was identified that is predicted to contain a 53 amino-acids C-terminus domain that is a homologue of the CHMP3 core domain [Hochstein *et al.*, 2016]. Thus, it was suggested that this virus uses this domain to recruit other Cdv proteins for its release site. However, other archaeal viruses (including a pyramid-like one) inhibit the Cdv system expression, instead of stimulating its expression, and, thus, probably use a Cdv-independent mechanism for their release [Okutan *et al.*, 2013, León-Sobrino *et al.*, 2016].

References

[Dobro *et al.*, 2013] Dobro, M. J., Samson, R. Y., Yu, Z., McCullough, J., Ding, H. J., Chong, P. L.-G., Bell, S. D., & Jensen, G. J. (2013). Electron cryotomography of ESCRT assemblies and dividing Sulfolobus cells suggests that spiraling filaments are involved in membrane scission. *Mol. Biol. Cell,* **24**, 2319–2327.

[Ellen *et al.*, 2009] Ellen, A. F., Albers, S.-V., Huibers, W., Pitcher, A., Hobel, C. F., Schwarz, H., Folea, M., Schouten, S., Boekema, E. J., Poolman, B., & Driessen, A. J. M. (2009). Proteomic analysis of secreted membrane vesicles of archaeal Sulfolobus species reveals the presence of endosome sorting complex components. *Extremophiles,* **13**, 67.

[Hochstein *et al.*, 2016] Hochstein, R. A., Amenabar, M. J., Munson-McGee, J. H., Boyd, E. S., & Young, M. J. (2016). Acidianus tailed spindle virus: a new archaeal large tailed spindle virus discovered by culture-independent methods. *J. Virol.* **90**, 3458–3468.

[León-Sobrino *et al.*, 2016] León-Sobrino, C., Kot, W. P., & Garrett, R. A. (2016). Transcriptome changes in STSV2-infected Sulfolobus islandicus REY15A undergoing continuous CRISPR spacer acquisition. *Mol. Microbiol.* **99**, 719–728.

[Lindås *et al.*, 2008] Lindås, A.-C., Karlsson, E. A., Lindgren, M. T., Ettema, T. J., & Bernander, R. (2008). A unique cell division machinery in the Archaea. *Proc. Natl. Acad. Sci. U.S.A.* **105**, 18942–18946.

[Liu *et al.*, 2017] Liu, J., Gao, R., Li, C., Ni, J., Yang, Z., Zhang, Q., Chen, H., & Shen, Y. (2017). Functional assignment of multiple ESCRT-III homologs in cell division and budding in Sulfolobus islandicus. *Mol. Microbiol.* **105**, 540–553.

[Maaty *et al.*, 2006] Maaty, W. S., Ortmann, A. C., Dlakić, M., Schulstad, K., Hilmer, J. K., Liepold, L., Weidenheft, B., Khayat, R., Douglas, T., Young, M. J., *et al.* (2006). Characterization of the archaeal thermophile Sulfolobus turreted icosahedral virus validates an evolutionary link among double-stranded DNA viruses from all domains of life. *J. Virol.* **80**, 7625–7635.

[Maaty *et al.*, 2012] Maaty, W. S., Selvig, K., Ryder, S., Tarlykov, P., Hilmer, J. K., Heinemann, J., Steffens, J., Snyder, J. C., Ortmann, A. C., Movahed, N., *et al.* (2012). Proteomic analysis of Sulfolobus solfataricus during Sulfolobus turreted icosahedral virus infection. *J. Proteome Res.* **11**, 1420–1432.

[Makarova *et al.*, 2010] Makarova, K. S., Yutin, N., Bell, S. D., & Koonin, E. V. (2010). Evolution of diverse cell division and vesicle formation systems in Archaea. *Nat. Rev. Microbiol.* **8**, 731.

[Moriscot *et al.*, 2011] Moriscot, C., Gribaldo, S., Jault, J.-M., Krupovic, M., Arnaud, J., Jamin, M., Schoehn, G., Forterre, P., Weissenhorn, W., & Renesto, P. (2011). Crenarchaeal CdvA forms double-helical filaments containing DNA and interacts with ESCRT-III-like CdvB. *PLoS One,* **6**, e21921.

[Ng *et al.*, 2013] Ng, K.-H., Srinivas, V., Srinivasan, R., & Balasubramanian, M. (2013). The Nitrosopumilus maritimus CdvB, but not FtsZ, assembles into polymers. *Archaea,* **2013**, 1–10.

[Okutan *et al.*, 2013] Okutan, E., Deng, L., Mirlashari, S., Uldahl, K., Halim, M., Liu, C., Garrett, R. A., She, Q., & Peng, X. (2013). Novel insights into gene regulation of the rudivirus SIRV2 infecting Sulfolobus cells. *RNA Biol.* **10**, 875–885.

[Olver & Vidal, 2007] Olver, C. & Vidal, M. (2007). Proteomic analysis of secreted exosomes. *Subcell. Biochem.* **43**, 99–131.

[Ortmann *et al.*, 2008] Ortmann, A. C., Brumfield, S. K., Walther, J., McInnerney, K., Brouns, S. J. J., van de Werken, H. J. G., Bothner, B., Douglas, T., van de Oost, J., & Young, M. J. (2008). Transcriptome analysis of infection of the archaeon Sulfolobus solfataricus with Sulfolobus turreted icosahedral virus. *J. Virol.* **82**, 4874–4883.

[Pelve *et al.*, 2011] Pelve, E. A., Lindås, A.-C., Martens-Habbena, W., de la Torre, J. R., Stahl, D. A., & Bernander, R. (2011). Cdv-based cell division and cell cycle organization in the thaumarchaeon Nitrosopumilus maritimus. *Mol. Microbiol.* **82**, 555–566.

[Samson *et al.*, 2008] Samson, R. Y., Obita, T., Freund, S. M., Williams, R. L., & Bell, S. D. (2008). A role for the ESCRT system in cell division in archaea. *Science,* **322**, 1710–1713.

[Snyder *et al.*, 2013] Snyder, J. C., Samson, R. Y., Brumfield, S. K., Bell, S. D., & Young, M. J. (2013). Functional interplay between a virus and the ESCRT machinery in archaea. *Proc. Natl. Acad. Sci. U.S.A.* **110**, 10783–10787.

[Snyder & Young, 2011] Snyder, J. C. & Young, M. J. (2011). Potential role of cellular ESCRT proteins in the STIV life cycle. *Biochem. Soc. Trans.* **39**, 107–110.

[Spang *et al.*, 2015] Spang, A., Saw, J. H., Jørgensen, S. L., Zaremba-Niedzwiedzka, K., Martijn, J., Lind, A. E., van Eijk, R., Schleper, C., Guy, L., & Ettema, T. J. G. (2015). Complex archaea that bridge the gap between prokaryotes and eukaryotes. *Nature,* **521**, 173–179.

[Yang & Driessen, 2014] Yang, N. & Driessen, A. J. M. (2014). Deletion of cdvB paralogous genes of sulfolobus acidocaldarius impairs cell division. *Extremophiles,* **18**, 331–339.

[Yang *et al.*, 2012] Yang, Z., Vild, C., Ju, J., Zhang, X., Liu, J., Shen, J., Zhao, B., Lan, W., Gong, F., Liu, M., Cao, C., & Xu, Z. (2012). Structural basis of molecular recognition between ESCRT-III-like protein Vps60 and AAA-ATPase regulator Vta1 in the multivesicular body pathway. *J. Biol. Chem.* **287**, 43899–43908.

[Zaremba-Niedzwiedzka *et al.*, 2017] Zaremba-Niedzwiedzka, K., Caceres, E. F., Saw, J. H., Bäckström, D., Juzokaite, L., Vancaester, E., Seitz, K. W., Anantharaman, K., Starnawski, P., Kjeldsen, K. U., Stott, M. B., Nunoura, T., Banfield, J. F., Schramm, A., Baker, B. J., Spang, A., & Ettema, T. J. G. (2017). Asgard archaea illuminate the origin of eukaryotic cellular complexity. *Nature,* **541**, 353–358.

Phylum<br>Order<br>Species	Property	CdvA	CdvB	CdvC
**Crenarchaeota**<br>Sulfolobales				
S. acidocaldarius	Old locus tag (aa length)	Saci_1374 (238)	Saci_1373 (261)	Saci_1372 (374)
	Division [Samson *et al.*, 2008, Lindås *et al.*, 2008]	+	+	+
	Vesicle Formation [Ellen *et al.*, 2009]			+
	Polymer formation [Dobro *et al.*, 2013]	+		
S. solfataricus	Old locus tag (aa length)	Sso_0911(238)	Sso_0910 (259)	Sso_0909 (372)
	Homology	238/65%/84%	260/64%/83%	375/72%/84%
	Vesicle Formation [Ellen *et al.*, 2009]			+
	Virus Budding [Ortmann *et al.*, 2008]	+	+	+
S. islandicus	Old locus tag (aa length)	SiRe_1173 (265)	SiRe_1174 (259)	SiRe_1175 (372)
	Homology	238/65%/84%	260/64%/83%	375/73%/85%
M. sedula	Old locus tag (aa length)	Msed_1670 (239)	Msed_1671 (270)	Msed_1672 (369)
	Homology	238/56%/78%	270/54%/74%	375/73%/86%
	Polymers [Moriscot *et al.*, 2011]	+	+	
Desulforococcales				
S. marinus	Old locus tag(aa length)	Smar_1276 (230)	Smar_1277 (260)[•]	Smar_1278 (379)
	Homology (aa length)	237/30%/62%	228/28%/45%	380/49%/67%
**Thaumarchaeota**<br>Thermoprotei				
N. Maritimus	Old locus tag (aa length)	Nmar_0700 (292)[†]	Nmar_1090 (186)[†*]	Nmar_1088 (397)[†]
	Homology	159/26%/52%	16/38%/62%	390/51%/70%
	Division [Pelve *et al.*, 2011]	+		+
**Lokiarchaeota**<br>Lokiarchaeota				
Lokiarchaeum sp.	Locus tag (aa length)<br>[Zaremba-Niedzwiedzka *et al.*, 2017]	-	Lokiarch_37480 (209)<br>Lokiarch_16760 (218)[‡]	Lokiarch_37470 (405)
	Homology		128/28%/40% 70/20%/47%	403/33%/53%

Table S1: **Summary of literature finding related to the main operon of the Cdv proteins.** Summary of the biological functions of the main Cdv locus proteins from several archaeal species. For consistency with published literature, proteins are annotated according to their NCBI old locus tag (except for *Lokiarchaeum sp.*). Homology values relative to *S. acidocaldarius* proteins (Length of homology region in amino acids / percentage of identical amino acids in this region / percentage of similar amino acids residues in this region) of several of these proteins are given based on the blast results (see Methods). References are given for each finding. Abbreviations: *S. acidocaldarius* - *Sulfolobus acidocaldarius DSM 639*; *S. solfataricus* - *Sulfolobus solfataricus P2*; *S. islandicus* - *Sulfolobus islandicus REY15A*; *M. sedula* - *Metallosphaera sedula DSM 5348*; *S. marinus* - *Staphylothermus marinus F1*; *N. Maritimus* - *Nitrosopumilus Maritimus SCM1*; *Lokiarchaeum sp.* - *Lokiarchaeum sp. GC14_75*. Notes: [†] Discontinued by NCBI. [•] Smar_0481, a CdvB homologue that is located outside of the Cdv main locus in *S. marinus, has* a higher homology to Saci_1372 than Smar_1277 that is located on the main Cdv locus in this organism, see Table S2.[‡] Lokiarch_16760 is in fact, a homologue of the Snf7 family of ESCRT-III proteins. See Fig.2. [*] Nmar_1090 belongs to a distinct branch of the Cdv proteins. See Fig.2.

Phylum / Order / Species	Property	CdvB1	CdvB2	CdvB3
**Crenarchaeota**				
Sulfolobales				
S. acidocaldarius	Old Locus tag (aa length)	Saci_0451(214)	Saci_1416 (219)	Saci_1601 (169)
	Division [Yang & Driessen, 2014]			+
	Vesicle formation [Ellen *et al.*, 2009]	+	+	
S. solfataricus	Old locus tag (aa length)	Sso_0451 (253)	Sso_0881 (221)	Sso_0619 (168)
	Homology	213/71%/84%	218/77%/90%	164/36%/60%
	Vesicle Formation [Ellen *et al.*, 2009]	+	+	+
	Virus Budding [Ortmann *et al.*, 2008]	+	+	+
S. islandicus	Old locus tag (aa length)	SiRe_1550 (253)	SiRe_1200 (221)	SiRe_1388 (168)
	Homology	212/71%/84%	218/77%/90%	167/37%/59%
	Division [Liu *et al.*, 2017]	+	+	
	Virus Budding [Liu *et al.*, 2017]			+
M. sedula	Old locus tag (aa length)	Msed_2179 (211)	Msed_1695 (221)	Msed_1969 (165)
	Homology	210/61%/79%	216/66%/85%	155/32%/59%
Desulforococcales				
S. marinus	Old locus tag (aa length)	Smar_0481 (212)		
	Homology	210/36%/80%		
**Thaumarchaeota**				
Thermoprotei				
N. Maritimus	Old locus tag (aa length) #	Nmar_0061(216)‡	Nmar_0816 (216)‡	Nmar_0029 (186)‡
	Homology	199/36%/61%	203/31%/54%	179/39%/60%
	Division [Pelve *et al.*, 2011]		?	?
	Polymers [Ng *et al.*, 2013]		+	
		ESCRT-I (Vps28)	ESCRT-II (Vps25)	
**Lokiarchaeota**				
Lokiarchaeota				
Lokiarchaeum sp.	Locus tag (aa length) [Zaremba-Niedzwiedzka *et al.*, 2017]	Lokiarch_10170 (218)	Lokiarch_37460 (297)	
	Homology	134/30%/47%	79/33%/54%⊙	

Table S2: **Summary of literature finding related to *S. acidocaldarius* CdvB proteins outside of the main Cdv operon.** Biological functions of CdvB paralogues from outside of the main Cdv locus. Annotation, methods, and functions - similar to table S1. Homology for *Lokiarchaeum sp.* ESCRT-I and ESCRT-II proteins are relative to *S. cerevisiae* Vps25 and Vps28 proteins. Notes: ⊙ The *Lokiarchaeum sp.* hypothetical ESCRT-II protein includes additional two short peptides (15 and 11 aa) with homology of (0.4/0.53 and 0.36/0.54) to Vps25. ‡ - Discontinued by NCBI. # Numbers are all relative to Saci_1416 since all three *N. Maritimus* CdvB paralogs belong to a distinct branch of the Cdv phylogenetic tree. See Fig.2. ? denotes an uncertain function.

Order / Species	Property	CdvB	CdvB'	CdvC
Thermococcales				
Thermococcus barophilus MP	Locus tag (aa length)	ZP_04876926 (201)	ZP_04876642 (198)	ZP_04876711 (393)
	Old locus tag	TERMP_1634	TERMP_1350	TERMP_1419
	Homology (CdvB/CdvC)	16/44%/75%; 31/35%/58%	20/35%/50% †	330/32%/52%
	Homology (CdvB1)	-	156/22%/39%	
	Homology (CdvB2)	17/355/645	129/28%/44%	
	Homology (CdvB3)	-	-	
Methanococcales				
Methanocaldococcus vulcanius M7	Locus tag (aa length)	ACX72860 (202)	ACX72851 (207)	ACX72859 (401)
	Old locus tag	Metvu_1002	Metvu_0993	Metvu_1001
	Homology (CdvB/CdvC)	56/27%/50%	90/26%/52%	292/38%/56%
	Homology (CdvB1)	83/19%/50%; 9/44%/88%	60/22%/43%	
	Homology (CdvB2)	99/30%/50%; 9/56%/77%	130/25%/50%	
	Homology (CdvB3)	20/40%/65%	-	
Thermoplasmatales				
Thermoplasma acidophilum DSM 1728	Locus tag (aa length)	WP_010901588 (212)		WP_010901582 (375)
	Old locus tag	Ta1181		Ta_1175
	Homology (CdvB/CdvC)	38/25%/52%		276/38%/58%
	Homology (CdvB1)	-		
	Homology (CdvB2)	-		
	Homology (CdvB3)	-		
Halobacteriales				
Halomicrobium_mukohataei_DSM_12286	Locus tag (aa length)	ACV49359 (214)	ACV49360 (222)	ACV49358 (412)
	Old locus tag	Hmuk_3259	Hmuk_3260	Hmuk_3258
	Homology (CdvB/CdvC)	93/26%/41%*	48/21%/50%$^{\oplus}$	182/40%/60%
	Homology (CdvB1)	-	-	
	Homology (CdvB2)	-	60/26%/43%	
	Homology (CdvB3)	95/26%/40%	-	

Table S3: **Characteristics of Cdv proteins orthologs from the Euryarchaeota phylum.** Several orthologues proteins of CdvB and CdvC that were identified in Euryarchaeota (see ref [Makarova *et al.*, 2010]). Annotation and homology parameters (relative to *S. acidocaldarius)* are the same as in table S1. Note: † - Homology only in the wH region. * - Homology between the $\alpha 5$ and the wH motifs. $^{\oplus}$ - Homology between $\alpha 4$ and $\alpha 5$.

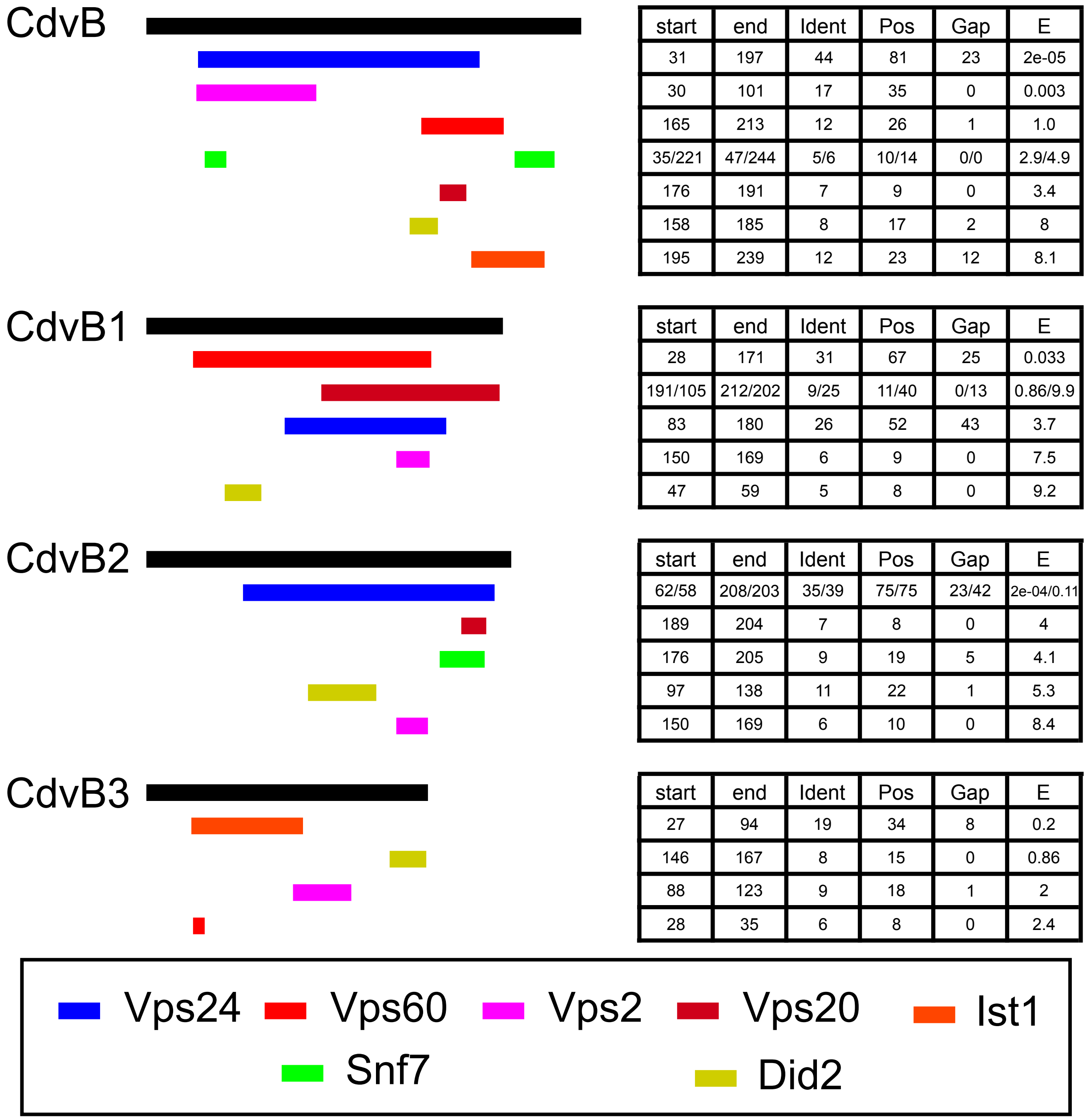

start	end	Ident	Pos	Gap	E
31	197	44	81	23	2e-05
30	101	17	35	0	0.003
165	213	12	26	1	1.0
35/221	47/244	5/6	10/14	0/0	2.9/4.9
176	191	7	9	0	3.4
158	185	8	17	2	8
195	239	12	23	12	8.1

start	end	Ident	Pos	Gap	E
28	171	31	67	25	0.033
191/105	212/202	9/25	11/40	0/13	0.86/9.9
83	180	26	52	43	3.7
150	169	6	9	0	7.5
47	59	5	8	0	9.2

start	end	Ident	Pos	Gap	E
62/58	208/203	35/39	75/75	23/42	2e-04/0.11
189	204	7	8	0	4
176	205	9	19	5	4.1
97	138	11	22	1	5.3
150	169	6	10	0	8.4

start	end	Ident	Pos	Gap	E
27	94	19	34	8	0.2
146	167	8	15	0	0.86
88	123	9	18	1	2
28	35	6	8	0	2.4

Figure S1: **Homology of CdvB proteins and the Yeast ESCRT-III proteins.** Homology areas and homology measured of the four *S. acidocaldarius* CdvB proteins vs. the ESCRT-III proteins of *S. cerevisiae* according to the color-coded legend. Start - Position of the first amino acid of the homology area in *S. acidocaldarius*. End - Position of the last amino acid in the homology region in *S. acidocaldarius*. Iden - Number of identical amino acids hits. Pos - Number of positive amino acids hits. Gap - Number of amino acids gaps in the homology area. E - blast E-values (calculated for blast of the relevant protein relative the group of all *S. cerevisiae* ESCRT-III proteins).
